# Multimodal channel cancer chemotherapy by 2D functional gadolinium metal–organic framework

**DOI:** 10.1093/nsr/nwaa221

**Published:** 2020-09-03

**Authors:** Jiale Xia, Yumeng Xue, Bo Lei, Lingling Xu, Mingzi Sun, Na Li, Hongyang Zhao, Min Wang, Meng Luo, Chao Zhang, Bolong Huang, Yaping Du, Chun-Hua Yan

**Affiliations:** Frontier Institute of Science and Technology, Xi’an Jiaotong University, Xi’an 710000, China; Frontier Institute of Science and Technology, Xi’an Jiaotong University, Xi’an 710000, China; Frontier Institute of Science and Technology, Xi’an Jiaotong University, Xi’an 710000, China; Frontier Institute of Science and Technology, Xi’an Jiaotong University, Xi’an 710000, China; Department of Applied Biology and Chemical Technology, The Hong Kong Polytechnic University, Hong Kong, China; Frontier Institute of Science and Technology, Xi’an Jiaotong University, Xi’an 710000, China; Frontier Institute of Science and Technology, Xi’an Jiaotong University, Xi’an 710000, China; Frontier Institute of Science and Technology, Xi’an Jiaotong University, Xi’an 710000, China; Frontier Institute of Science and Technology, Xi’an Jiaotong University, Xi’an 710000, China; Tianjin Key Lab for Rare Earth Materials and Applications, School of Materials Science and Engineering, National Institute for Advanced Materials, Center for Rare Earth and Inorganic Functional Materials, Nankai University, Tianjin 300350, China; Department of Applied Biology and Chemical Technology, The Hong Kong Polytechnic University, Hong Kong, China; Frontier Institute of Science and Technology, Xi’an Jiaotong University, Xi’an 710000, China; Tianjin Key Lab for Rare Earth Materials and Applications, School of Materials Science and Engineering, National Institute for Advanced Materials, Center for Rare Earth and Inorganic Functional Materials, Nankai University, Tianjin 300350, China; Tianjin Key Lab for Rare Earth Materials and Applications, School of Materials Science and Engineering, National Institute for Advanced Materials, Center for Rare Earth and Inorganic Functional Materials, Nankai University, Tianjin 300350, China; Beijing National Laboratory for Molecular Sciences, State Key Laboratory of Rare Earth Materials Chemistry and Applications, PKU–HKU Joint Laboratory in Rare Earth Materials and Bioinorganic Chemistry, College of Chemistry and Molecular Engineering, Peking University, Beijing 100871, China; College of Chemistry and Chemical Engineering, Lanzhou University, Lanzhou 730000, China

**Keywords:** lanthanide-based porphyrin metal–organic framework, drug delivery, stimuli-responsive degradation/release, tumor tissue penetration, cancer chemotherapy

## Abstract

2D nanomaterials generally exhibit enhanced physiochemical and biological functions in biomedical applications due to their high surface-to-volume ratio and surface charge. Conventional cancer chemotherapy based on nanomaterials has been hindered by their low drug loading and poor penetration in tumor tissue. To overcome these difficulties, novel materials systems are urgently needed. Hereby, the lanthanide-based porphyrin metal–organic framework (MOF) nanosheets (NSs) with promising cancer imaging/chemotherapy capacities are fabricated, which display superior performance in the drug loading and tumor tissue penetration. The biodegradable PPF-Gd NSs deliver an ultrahigh drug loading (>1500%) and demonstrate the stable and highly sensitive stimuli-responsive degradation/release for multimodal tumor imaging and cancer chemotherapy. Meanwhile, PPF-Gd NSs also exhibit excellent fluorescence and magnetic resonance imaging capability *in vitro* and *in vivo*. Compared to the traditional doxorubicin (DOX) chemotherapy, the *in vivo* results confirm the evident suppression of the tumor growth by the PPF-Gd/DOX drug delivery system with negligible side effects. This work further supports the potential of lanthanide-based MOF nanomaterials as biodegradable systems to promote the cancer theranostics technology development in the future.

## Teaser

The unique gadolinium MOF based nanosheets with the paddlewheel frameworks have been constructed to realize the ultrahigh drug loading and bimodal-imaging guided cancer chemotherapy.

## INTRODUCTION

Recently, 2D materials, such as graphene [1–3], black phosphorus [4], MXenes [5,6], transition metal dichalcogenides [7] and hexagonal boron nitride [8], have gained tremendous research interest due to their distinct chemical/physical properties and remarkable surface properties compared with their bulk counterparts. Among the various types of 2D materials, 2D metal–organic framework (MOF) nanosheets (NSs), which are constructed by metallic nodes and organic ligands through coordination bonds, deliver many applications such as gas separation, storage, molecular recognition and drug delivery, benefiting from their tunable pores and cavities [9–11]. Porphyrin molecules are remarkable candidates as the building blocks for MOF construction due to their high thermal/chemical stabilities and facile synthesis [12,13]. More importantly, porphyrin molecules, as a class of stable organic chromophores, can efficiently absorb visible light and emit fluorescence (FL), which reveal their potential applications in many biological functions in aqueous media, such as oxygen transportation, light harvesting, FL labeling and cancer therapy [14–16].

Over the past few decades, cancer has become one of the most life-threatening diseases for humans. However, many conventional effective drugs still face challenges such as hydrophobicity, fast metabolism and toxic side effects, leading to decreased efficiencies [17]. One of the most promising ways to address these issues is to employ a drug delivery system to improve drug efficacy through enhanced delivery, targeting, protection and controllable drug release [9,18]. Nonetheless, on one hand, conventional drug delivery platforms are facing key challenges including low drug loading and poor penetration in tumor tissue [19]. On the other hand, bioimaging technologies, such as X-ray computed tomography, magnetic resonance imaging (MRI), ultrasound imaging and FL imaging, are indispensable tools for disease diagnosis and therapy [20–24]. In recent years, more and more researchers have paid attention to novel multifunctional cancer therapy platforms combining with imaging systems. Yang's group developed a near-infrared cancer therapy where a nanomicelle encapsulates a fluorescent probe to enable the real-time visualized and precise cancer therapy [25]. Zhang and coworkers synthesized Au-coated Fe_3_O_4_ core–shell nanoparticles to serve as an excellent theranostic agent for efficient MRI-guided cancer diagnosis and treatment [26]. The imaging-guided cancer therapy systems make the visualized cancer therapy possible, thus significantly increasing the therapy efficiency and reducing the side effects. However, single imaging modalities often face problems such as inadequate sensitivity and poor spatial resolution, making it difficult to collect precise clinical information and reliable diagnostic results [27,28]. With the combination of different imaging modalities, more accurate and visible information can be easily obtained. Under these circumstances, the development of novel and highly efficient anticancer medical strategies combined with multiple bioimaging diagnostics for cancer therapy is urgently needed.

Owing to the unique 4f orbitals of lanthanide elements, lanthanide-based functional nanomaterials exhibit significant magnetic and luminescence properties, thereby making them serve as promising candidates for diagnostic and therapeutic applications, including biosensing, bioimaging and photodynamic/photothermal therapy [29–32]. In particular, all Ln^3+^ ions (except for La^3+^ and Lu^3+^) have unpaired electrons in 4f orbitals, which thus induce significant paramagnetic properties [33,34]. Among these Ln^3+^ ions, Gd^3+^ has the largest number of unpaired electrons and relatively long electronic relaxation time, which makes Gd^3+^ the most efficient *T*_1_ relaxation enhancement [35,36]. In addition, the magnetic properties of Gd^3+^ are not markedly influenced by coordinated ligand because of the shielded 4f orbitals from the ligand, indicating excellent magnetic properties of Gd_2_O_3_, Gd^3+^-doped material or Gd^3+^ coordination compounds [37–41], thereby allowing Gd^3+^-based nanomaterials to serve as outstanding MR contrast agents.

**Figure 1.**
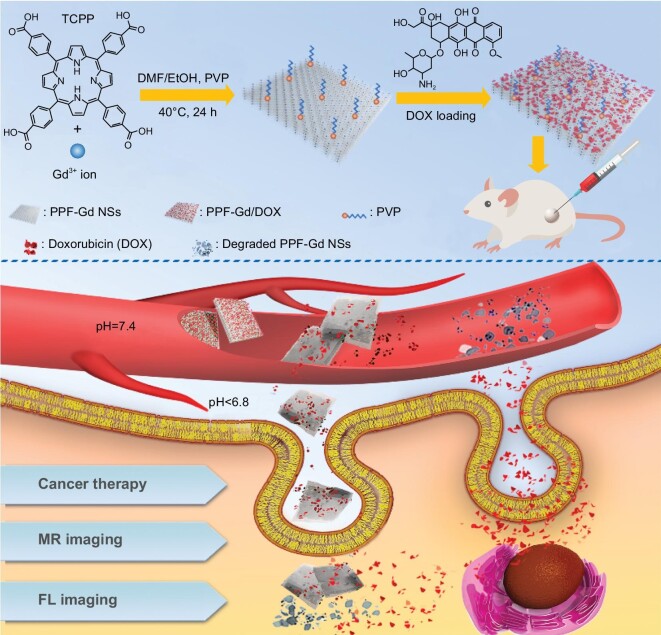
Schematic illustration of the fabrication of PPF-Gd NS- and PPF-Gd NS-based drug delivery systems toward efficient cancer therapy and multimodal bioimaging.

Herein, we constructed a series of lanthanide (Ln = Ce, Eu, Gd, Tb and Ho) porphyrin MOF NSs (labeled as PPF-Ln) with a surfactant-assisted wet chemistry strategy (Fig. S1). Among all these NSs, the multifunctional gadolinium-based porphyrin paddlewheel framework (PPF-Gd) was chosen for further cancer imaging and therapy. PPF-Gd nanomaterials (Figs S2 and S3) and micromaterials (Fig. S4) with controllable thicknesses and lateral sizes were synthesized via a surfactant-assisted solvothermal method. The fabrication and cancer theranostics application of PPF-Gd NSs are depicted in Fig. 1. We employed tetrakis(4-carboxyphenyl)porphyrin (TCPP) as the organic ligand and gadolinium as the bridging metal ion. The square planar symmetry and diverging carboxylic groups of TCPP make it easy to self-assemble to form quadrangular networks [42,43]. More importantly, TCPP shows excellent photoluminescence properties, and the isolated porphyrin ligands can prevent FL from self-quenching [44]. Meanwhile, gadolinium is the most effective and extensively used contrast element for MRI [45,46]. The excellent photoluminescence and magnetic properties endow the PPF-Gd NSs with high-quality FL and MRI capabilities. In addition, the extraordinary surface area of PPF-Gd NSs due to their high porosity and 2D morphology enables the highly efficient loading of therapeutic drugs such as doxorubicin (DOX) presented in this study. The unique properties of the obtained PPF-Gd NSs make it a very promising drug delivery vehicle and a bimodal imaging probe for cancer therapy.

## RESULTS AND DISCUSSION

### Synthesis and characterization of PPF-Gd

**Figure 2.**
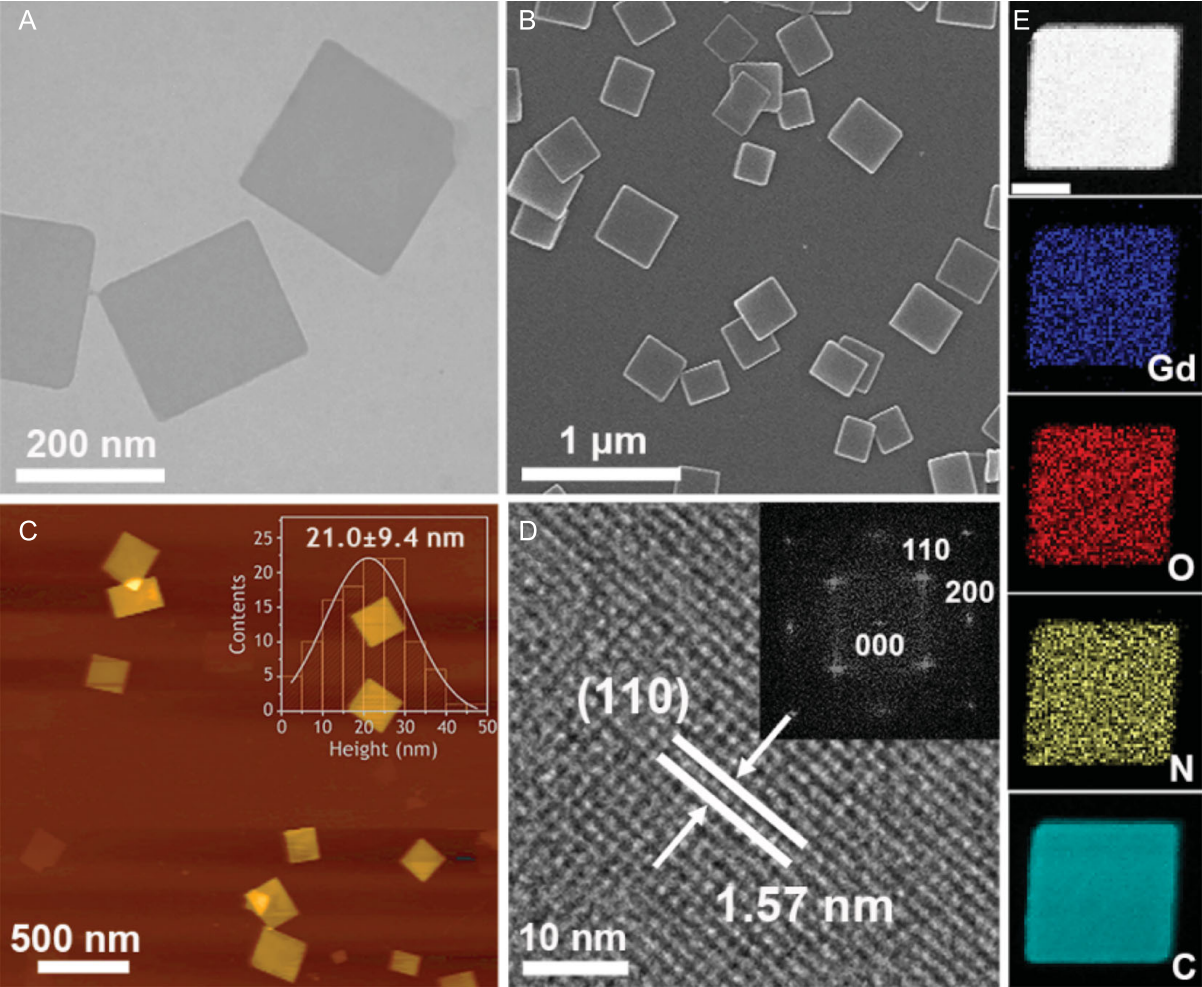
Structural characterization of PPF-Gd NSs. (A) TEM image and (B) SEM image of the PPF-Gd NSs. (C) AFM image and statistical analysis of the thickness of PPF-Gd NSs as measured by AFM. (D) STEM image of PPF-Gd NSs with the FFT image (inset). (E) STEM-EELS elemental mapping images of elements Gd, C, N and O. Scale bar = 100 nm.

The obtained PPF-Gd NSs were characterized by transmission electron microscopy (TEM), scanning transmission electron microscopy (STEM), scanning electron microscopy (SEM), atomic force microscopy (AFM) and powder X-ray diffraction (XRD). PPF-Gd NSs show a square-like morphology with a length of 221 ± 63 nm and a thickness of 21.0 ± 9.4 nm (Figs 2A–C and S2). The STEM image shows a lattice fringe with an interplanar distance of 1.57 nm (Fig. 2D), which can be ascribed to the (110) plane of PPF-Gd crystal. The corresponding fast Fourier transform (FFT) analysis reveals a monoclinic crystal structure (inset in Fig. 2D), and the diffraction spots are attributed to the (110) and (200) planes of PPF-Gd NSs, which is in good agreement with the XRD results (Fig. S5). In addition, the electron energy loss spectroscopy (EELS) mapping images of PPF-Gd NSs show uniform distribution of C, N, O and Gd elements in the entire NS, which is consistent with the X-ray photoelectron spectroscopy results (Fig. S6).

### Drug loading and release ability of PPF-Gd

**Figure 3.**
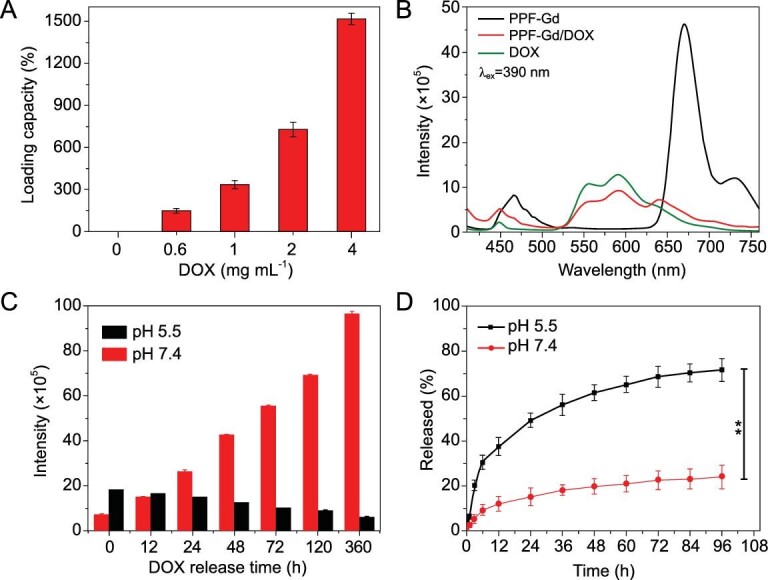
Drug loading and release capability of PPF-Gd. (A) DOX loading capacity of PPF-Gd at various DOX concentrations. (B) The FL spectra of PPF-Gd NSs, PPF-Gd/DOX (300% DOX loading capacity) and DOX under 390 nm excitation (0.03 M in terms of PPF-Gd NSs). (C) The FL intensity of PPF-Gd/DOX at pH 5.5 and 7.4 at different release times under 390 nm excitation. (D) DOX released from PPF-Gd/DOX at pH 5.5 and 7.4 versus time.

The Brunauer–Emmett–Teller surface area and pore size of PPF-Gd NSs were calculated to be 325.64 m^2^ g^−1^ and 35.4 Å, respectively (Fig. S7). Considering the high surface area as well as the large channel voids (∼5 Å) [42], the PPF-Gd NSs are a promising candidate as a superb carrier for anticancer drugs. In addition, the PPF-Gd NSs are negatively charged in water and therefore may capture the small positively charged drug molecules into their interlayer spaces. In order to investigate the drug loading ability of PPF-Gd NSs, we chose DOX as the model drug since it is widely used in cancer chemotherapy. The DOX loading capacity was assessed by UV–vis spectroscopy, and the absorption peak at 480 nm was used to determine the successful loading of DOX on PPF-Gd NSs (Fig. S8). With an increase in DOX concentrations, the loading capacity was increased and reached 1515% when the DOX concentration was 4 mg mL^−1^, which is much higher than the reported value to date (Fig. 3A and Table S1) [47,48]. In addition, the zeta potential values increased from −11.3 to +18.8 mV with the increased DOX concentrations (Fig. S9). The ultrahigh drug loading capacity can be ascribed to the collective effect of the large surface area, electrostatic interactions and strong interactions such as hydrogen bonding or π–π conjugation between PPF-Gd NSs and DOX.

In addition, the DOX loading and release could be monitored by measuring the FL of PPF-Gd NSs, DOX and PPF-Gd/DOX (Fig. 3B). PPF-Gd NSs show the main emission peak at 670 nm and two small peaks at 467 and 731 nm when excited at 390 nm. Meanwhile, DOX shows a small emission peak at 449 nm and two main peaks at 557 and 592 nm. Interestingly, after DOX loading, the FL of PPF-Gd NSs was significantly quenched, suggesting the strong interactions between PPF-Gd NSs and DOX. As DOX released, the emission at 670 nm characteristic of PPF-Gd NSs was gradually recovered, which could be used to monitor the drug release process (Figs 3C and  S10). Specifically, it can be observed that the FL intensity at 664 nm increased rapidly with the release of DOX at pH 7.4. However, the FL intensity gradually decreased with DOX release at pH 5.5, which could be attributed to the structural disintegration of PPF-Gd NSs under acidic environment.

The stimuli-responsive drug release behaviors of PPF-Gd NSs were then studied in phosphate-buffered saline (PBS) solutions at pH 7.4 and 5.5, respectively (Fig. 3D). At pH 5.5, ∼49% DOX was released over 24 h, while only 15% DOX was released over 24 h at pH 7.4, confirming the pH-responsive drug release behavior. The release of DOX gradually increased, and finally reached 72% and 24% after 96 h at pH 5.5 and 7.4, respectively. The faster release kinetics at pH 5.5 was probably due to the protonation of the amino group in DOX and weakened hydrogen bond between PPF-Gd NSs and DOX. The pH-responsive drug release behavior benefits the application for intratumoral drug delivery due to the acidic microenvironment of tumor. More importantly, PPF-Gd NSs show a pH-responsive biodegradation property. The structure of PPF-Gd NSs was etched into porous NSs at pH 5.5 for 12 h, while the structure remained stable at pH 7.4. After 72 h, PPF-Gd NSs completely degraded into small nanoparticles at pH 5.5, while the square-like structure was basically retained in a PBS solution at pH 7.4 (Fig. S11). The pH-responsive biodegradation enables controlled release of therapeutic drugs from PPF-Gd NSs as well as the clearance of the empty carrier after drugs are delivered to targeted cells.

### Dispersion stability of PPF-Gd NSs

**Figure 4.**
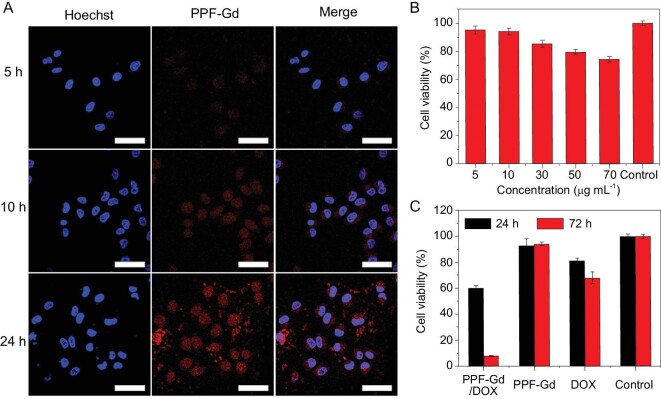
(A) Confocal images of A375 cells incubated with PPF-Gd NSs for 5, 10 and 24 h, respectively. Scale bar = 50 μm. (B) The viability of C2C12 cells in the presence of PPF-Gd NSs at different concentrations. (C) The viability of A375 cells in the presence of PPF-Gd/DOX, PPF-Gd NSs and DOX (50 μg mL^−1^ in terms of PPF-Gd NSs).

The dispersion stability of nanomaterials in the physiological environment was very important for their *in vivo* biomedical applications. The PPF-Gd NSs could be readily dispersed in water, PBS and serum medium. After 12 h, there was a slight sedimentation in PBS solution, while there was no agglomeration in water and serum medium (Fig. S12), indicating their remarkable physiological stability. The cellular uptake of PPF-Gd NSs was investigated by incubating them with A375 cancer cells for 5, 10 and 24 h, respectively (Fig. 4A). At 5 h, the weak red FL signal in cytoplasm indicated the fast cellular uptake of PPF-Gd NSs. An obviously increased FL signal over incubation time was observed in nuclei, demonstrating the transportation of PPF-Gd NSs from the cytoplasm into cell nuclei. The results suggested that PPF-Gd NSs can serve as a desirable carrier for delivering chemotherapeutic drugs into the nuclei of cancer cells.

### Hemocompatibility analysis of PPF-Gd

Then, the hemocompatibility analysis of PPF-Gd NSs showed that PPF-Gd NSs exhibited relatively low hemolysis (below 10%) when the concentration was up to 125 μg mL^−1^ and there was no obvious change of the cell morphology after the treatment of NSs, which indicated the excellent hemocompatibility of the NSs (Fig. S13). Subsequently, the cellular biocompatibility of PPF-Gd NSs was evaluated using myoblasts (C2C12 cells) at different concentrations (Fig. 4B) to further study the biocompatibility. PPF-Gd NSs exhibited dose-dependent cytotoxicity, and the viability was ∼94% when the concentration was 10 μg mL^−1^. Besides, the viability was still above 70% even when the concentration was as high as 70 μg mL^−1^, which is far beyond the proper concentration for *in vivo* experiments, indicating the low cytotoxicity of PPF-Gd NSs. Figure 4C shows the A375 cancer cell-killing capability of PPF-Gd/DOX, PPF-Gd NSs and DOX in terms of 50 μg mL^−1^ PPF-Gd. It was clear that PPF-Gd/DOX had the highest cell-killing capability, which can be ascribed to the high loading and slow but persistent DOX release from PPF-Gd NSs. After 72 h, PPF-Gd/DOX showed significantly higher toxicity for cancer cells (only 10% cell viability left), as compared to DOX (65% cell viability) at the same dose. The drug delivery system can significantly improve the therapeutic effect and reduce the DOX dose at the same time, especially for the long-term *in vivo* therapy experiment.

The pharmacokinetics of PPF-Gd NSs was also conducted to investigate the biosafety of the NSs. The analysis of Gd amount in different tissues at different treatment times confirmed that PPF-Gd was gradually decreased in various organs as a function of time and PPF-Gd NSs were effectively cleared through the liver and spleen (Fig. S14), which indicated the gradual degradation behavior and the low *in vivo* toxicity of PPF-Gd NSs.

### Permeability and FL imaging ability of the PPF-Gd NSs

**Figure 5.**
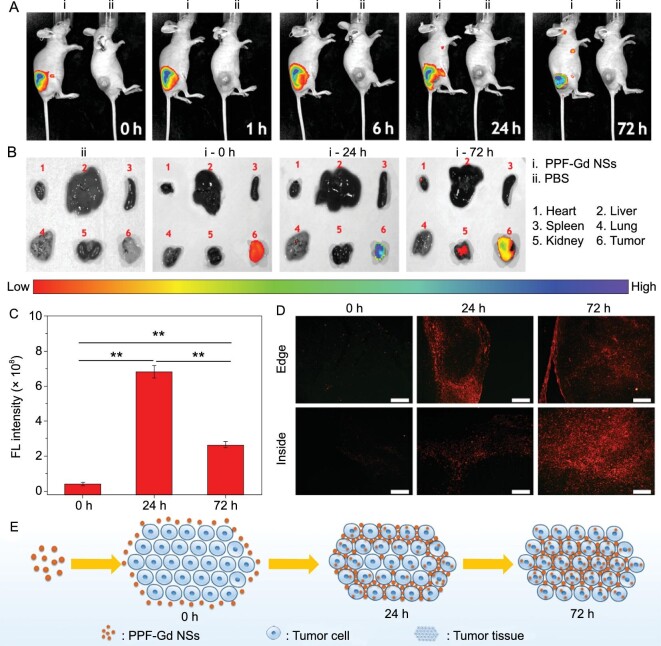
FL imaging. (A) *In vivo* FL imaging and biodistribution of nude mice bearing A375 tumors at different time points after subcutaneous injection of PPF-Gd NSs and PBS in the subcutaneous area around tumor tissue. (B) FL imaging and distribution of PPF-Gd NSs in major organs of nude mice after subcutaneous injection around tumor tissue for 0, 24 and 72 h, respectively. (C) Quantitative analysis of FL intensity of tumors at different time points after PPF-Gd NS injection (^**^P < 0.01). (D) FL imaging and intratumoral microdistribution of PPF-Gd NSs at the edge and inside area of tumor tissues collected at different time points (PPF-Gd NSs: red FL; scale bar = 200 μm). (E) Schematic illustration of the microdistribution of PPF-Gd NSs in tumor tissue after subcutaneous injection around tumor tissue.

Bioimaging is an indispensable tool for non-invasive diagnosis during cancer therapy. As the FL signal of PPF-Gd NSs is strong and stable enough to be observed under a confocal microscope (Fig. S15), PPF-Gd NSs were employed for *in vivo* FL imaging of the tumor through both intravenous and subcutaneous injection to study the permeability and FL imaging ability of the PPF-Gd NSs. After subcutaneous injection of PPF-Gd NSs around tumor tissues, the FL signal could be constantly monitored for >72 h, while no FL signal could be detected for the PBS control group, indicating the excellent capability for long-term *in vivo* tumor imaging (Fig. 5A). With the increase of time, the highest FL signal expanded from the injection point to the entire tumor tissue. The quantitative analysis based on the FL imaging of mice showed that the FL intensity of tumor tissue increased gradually in the first 24 h while decreased after 72 h, which may be attributed to the high penetration ability of PPF-Gd NSs into deep tumor tissue (Figs S17 and 5B–D). Major organs (heart, liver, spleen, lung and kidney) and tumors of nude mice were also detected for FL, in which significantly high FL intensity in tumor tissue could be observed at various time points (Fig. 5B and C). To verify our hypothesis, the tumor tissues were fixed and observed under the inverted FL microscope to investigate the intratumoral microdistribution of PPF-Gd NSs (red FL) (Fig. 5D). As we expected, no FL signal could be observed in tumor tissue after treatment with PPF-Gd NSs at first (0 h). After 24 h injection, enhanced red FL signals were detected at the tumor edge, while relatively weak FL signals were observed in the deeper tumor tissue areas. After 72 h, the strong red FL signal was distributed in the entire tumor tissue, suggesting the superior tumor penetration ability of PPF-Gd NSs. The outstanding tumor penetration ability of PPF-Gd NSs may be associated with the pH-responsive degradation property and excellent cell uptake ability of PPF-Gd NSs. After the injection, PPF-Gd NSs could gradually diffuse from the edge areas of tumor tissues to the inside areas. Combined with the study of degradation property of PPF-Gd NSs, PPF-Gd NSs would gradually degrade into small nanoparticles after exposure to tumor tissues (pH < 6.8), which could further promote the penetration and deposition of PPF-Gd NSs in deep tumor tissues (Fig. 5E). In the case of intravenous injection (Fig. S16), strong FL signals were observed immediately on the tail after intravenous injection. As time goes on, the FL intensity on the tail was fading, while the FL intensity among the body grew stronger. After 72 h injection, obvious FL signals can be observed in the tumor tissue, indicating that PPF-Gd NSs were gradually transferred to other parts of the body through the blood circulation and could effectively accumulate in tumor tissue by the enhanced permeability and retention (EPR) effect.

### MRI performance

**Figure 6.**
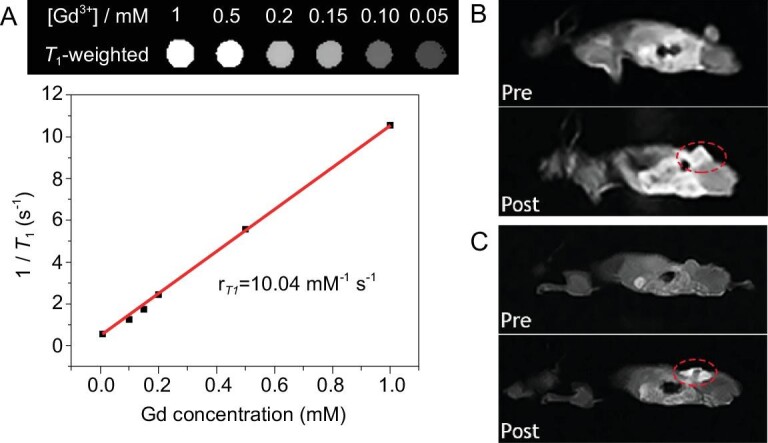
(A) Top: *T*_1_-weighted MR images of PPF-Gd NS solutions with different concentrations. Bottom: linear curve of relaxation rate (1/*T*_1_) versus the Gd^3+^ concentration. (B) *In vivo T*_1_-weighted MR coronal images of a nude mouse bearing A375 tumor before and after intravenous injection of PPF-Gd NSs. (C) *In vivo T*_1_-weighted MR coronal images of a nude mouse bearing A375 tumor before and after subcutaneous injection of PPF-Gd NSs.

Since gadolinium has unique magnetic properties and important diagnostic applications in MRI, the efficacy of PPF-Gd NSs for MRI was demonstrated. The *T*_1_-weighted MR signals of PPF-Gd NS solution revealed a concentration-dependent effect, and the increase in Gd^3+^ concentration led to the linear increase in the *T*_1_-weighted MR signal with a transverse relaxivity (*r*_1_) of 10.04 mM^−1^ s^−1^ (Fig. 6A). Both the intravenous and subcutaneous injection methods were employed for *in vivo* MRI measurement. After the intravenous injection for 6 h, the MR signal of the whole mouse grew stronger and the MR intensity of tumor tissue was significantly higher than that before treatment. This indicated that PPF-Gd could safely be circulated and accumulated at tumor tissue by the EPR effect (Fig. 6B). After injection in the tumor, the strong MR signal of PPF-Gd NSs could still be detected after 6 h, indicating the excellent tumor accumulation ability and permeability of the PPF-Gd NSs (Fig. 6C). These results suggested that PPF-Gd NSs can allow for effective FL/MR dual modal bioimaging, thereby making it possible to achieve both high spatial resolution and penetration depth for clinical diagnosis.

### *In vivo* therapeutic effect of drug delivery

**Figure 7.**
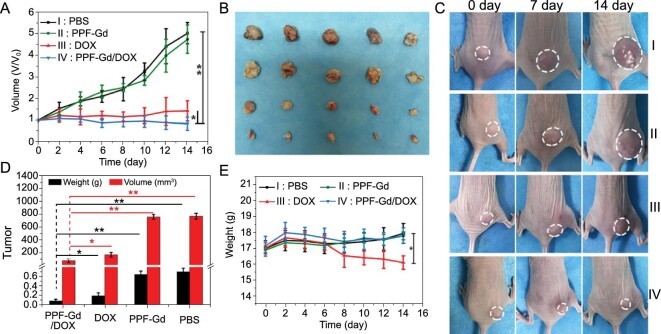
(A) Tumor growth curves of A375 tumor-bearing mice after different treatments through subcutaneous injection. The tumor volumes were normalized to the initial volumes. (B) Photographs of tumors from different groups after 14-day treatment. (C) Photographs of representative tumors in mice with different treatments. (D) Tumor weights and volumes of each group on the 14th day (^*^P < 0.05, ^**^P < 0.01). (E) The body weights of tumor-bearing mice measured every other day.

The *in vivo* therapeutic effect of PPF-Gd NS drug delivery system was investigated in the A375 tumor-bearing mouse model. After subcutaneous injection, the PPF-Gd/DOX and DOX groups could significantly inhibit the tumor growth, while the PBS and PPF-Gd groups exhibited no therapeutic effect (Fig. 7A–D). As expected, compared with the DOX group, the PPF-Gd/DOX group showed increased tumor inhibition efficacy, which was in good agreement with the *in vitro* results. Meanwhile, the weights of mice were slightly increased during the treatment in the PPF-Gd/DOX and PPF-Gd groups (Fig. 7E). However, the DOX group showed a gradual decline in body weights from the sixth day, indicating the negative side effects of DOX during the treatment. The H&E and TUNEL staining pictures further confirmed that the cancer cells showed significant apoptosis in the PPF-Gd/DOX and DOX groups (Fig. S18). The histological analyses of typical organs, including heart, liver, spleen, lung and kidney, of the mouse in each group are presented in Fig. S19. No obvious tissue toxicity was found in the PPF-Gd/DOX, PPF-Gd and PBS groups, while some morphological or pathological changes could be found in the DOX group, such as the atrophy of glomerulus in the kidney, inflammation of the liver and thickening of alveolar walls in the lung. It is worth mentioning that these four groups show similar *in vivo* treatment results through intravenous injection (Figs S20–S22). The histological analysis (H&E staining) of the main organs showed negligible side effects of PPF-Gd NSs during the intravenous treatment process. These results confirmed that PPF-Gd/DOX could be an excellent candidate in cancer therapy with enhanced therapeutic efficiency and low toxic side effects.

## CONCLUSION

In summary, we constructed a series of PPF-Ln NSs via a surfactant-assistant solvothermal method. Among these PPF-Ln nanomaterials, the multifunctional biodegradable 2D PPF-Gd NSs with an edge length of 221 ± 63 nm and a thickness of 21.0 ± 9.4 nm were used for tumor microenvironment-responsive drug delivery and bimodal imaging-guided cancer chemotherapy. PPF-Gd NSs showed ultrahigh anticancer drug (DOX) loading capacity (>1500%), pH-responsive biodegradation and persistent drug release, sustained tumor tissue imaging ability, enhanced tumor tissue penetration and significantly high tumor inhibition performance *in vitro* and *in vivo*. Our work suggests that biodegradable 2D nanomaterials with ultrahigh drug loading are promising for enhanced cancer imaging and chemotherapy.

## Supplementary Material

nwaa221_Supplemental_File
