# Supplementary material for: Multimodal channel cancer chemotherapy by 2D functional gadolinium metal–organic framework
Source: Natl Sci Rev. 2020 Sep 3;8(7):nwaa221. doi: 10.1093/nsr/nwaa221 (PMC8310757; doi:10.1093/nsr/nwaa221)
Supplement: nwaa221_Supplemental_File [file nwaa221_supplemental_file.docx]

Supporting Information

**Multi-modal Channel Cancer Chemotherapy by 2D Functional Gadolinium Metal-Organic Framework**

Jiale Xia^1,⊥^, Yumeng Xue^1,⊥^, Bo Lei^1,⊥^, Lingling Xu^1^, Mingzi Sun^3^, Na Li^1^, Hongyang Zhao^1^, Min Wang^1^, Meng Luo^1^, Chao Zhang^2^, Bolong Huang^3^*, Yaping Du^1,2^* and Chun-Hua Yan^2,4,5^

^1^ Frontier Institute of Science and Technology, Xi’an Jiaotong University, Xi’an 710000, China.

^2^ Tianjin Key Lab for Rare Earth Materials and Applications, School of Materials Science and Engineering, National Institute for Advanced Materials, Center for Rare Earth and Inorganic Functional Materials, Nankai University, Tianjin 300350, China.

Email: [ypdu@nankai.edu.cn](mailto:ypdu@nankai.edu.cn) (Prof. Y. Du).

^3^ Department of Applied Biology and Chemical Technology, The Hong Kong Polytechnic University, Hung Hom, Kowloon, Hong Kong SAR, China.

E-mail: [bhuang@polyu.edu.hk](mailto:bhuang@polyu.edu.hk) (Prof. B. Huang).

^4^ Beijing National Laboratory for Molecular Sciences, State Key Laboratory of Rare Earth Materials Chemistry and Applications, PKU-HKU Joint Laboratory in Rare Earth Materials and Bioinorganic Chemistry, College of Chemistry and Molecular Engineering, Peking University, Beijing 100871, China

^5^ College of Chemistry and Chemical Engineering, Lanzhou University, Lanzhou 730000, China

⊥These authors contributed equally to this work.

**Experimental Section**

**Materials.** Gadolinium nitrate hexahydrate (Gd(NO_3_)_3_·6H_2_O, 99.99%), cerium nitrate hexahydrate (Ce(NO_3_)_3_·6H_2_O, 99.99%), europium nitrate hexahydrate (Eu(NO_3_)_3_·6H_2_O, 99.99%), terbium nitrate hexahydrate (Tb(NO_3_)_3_·6H_2_O, 99.99%) and holmium nitrate pentahydrate (Ho(NO_3_)_3_·5H_2_O, 99.99%) were purchased from Beijing HWRK Chemical Company, Polyvinylpyrrolidone (PVP, average mol wt 40,000), N,N-Dimethylformamide (DMF, 98%), ethanol (EtOH) were purchased from Sigma-Aldrich. Tetrakis(4-carboxyphenyl)porphyrin (TCPP, 97%) was purchased from Tokyo Chemical Industry Co. Ltd. The deionized (DI) water was obtained from the Milli-Q System. All the materials were used without further purification.

**Characterizations.** The morphologies were characterized by a field emission scanning electron microscope (FE-SEM) (Zeiss Gemini SEM 500, German), Transmission electron microscopy (TEM) (Hitachi HT7700, Japan), Cs-corrected STEM (Hitachi 2700C, Japan) and atomic force microscopy (AFM) (Veeco INNOVA, USA). The crystal structure was characterized by X-ray diffraction (XRD) (Rigaku D/MAX-RB, Japan) using monochromatized Cu Kα radiation (λ=1.5418 Å). X-ray photoelectron spectra (XPS) were recorded using a PHI Quantera SXM instrument equipped with an Al X-ray excitation source (1486.6 eV).

**Synthesis of PPF-Ln materials (Ln = Ce, Eu, Gd, Tb and Ho)**

**Synthesis of 2D PPF-Gd nanosheets (PPF-Gd NSs).** A total of 6.77 mg Gd(NO_3_)_3_·6H_2_O and 50 mg PVP were dissolved in 12 mL mixture of DMF and EtOH (v:v=3:1) in a 20 mL capped glass vial. Then 4 mg TCPP dissolved in 4 mL mixture of DMF and EtOH (v:v=3:1) was added dropwise under stirring. After that, the vial was heated to 40 °C quickly and then kept for 24 h under continuous stirring. The resulting purple nanosheets were washed with DI water for 3-5 times and collected by centrifugation at 9500 rpm for 5 min. Finally, the obtained PPF-Gd NSs were freeze-dried.

**Synthesis of 2D PPF-Gd nanoplates.** A total of 13.54 mg Gd(NO_3_)_3_·6H_2_O and 100 mg PVP were dissolved in 12 mL mixture of DMF and EtOH (v:v=3:1) in a 20 mL capped glass vial. Then 8 mg TCPP dissolved in 4 mL mixture of DMF and EtOH (v:v=3:1) was added dropwise under stirring. After that, the solution was sonicated for 10 min. Then the solution was transferred into a 25 mL Teflon lined autoclave and heated to 90 °C and then kept the reaction for 24 h. The resulting purple nanoplates were washed with DI water for 3-5 times and collected by centrifugation at 9500 rpm for 5 min. Finally, the obtained PPF-Gd nanoplates were freeze-dried.

**Synthesis of PPF-Gd micro materials.** A total of 27.08 mg Gd(NO_3_)_3_·6H_2_O was dissolved in 12 mL mixture of DMF and EtOH (v:v=3:1) in a 20 mL capped glass vial. Then 16 mg TCPP dissolved in 4 mL mixture of DMF and EtOH (v:v=3:1) was added dropwise under stirring. After that, the solution was transferred into a 25 mL Teflon lined autoclave at 110 °C for 24 h. The resulting dark purple product were washed 3~5 times with DI water and collected by centrifugation at 9500 rpm for 5 min. Finally, the obtained PPF-Gd bulk materials were freeze-dried.

**Synthesis of PPF-Ln nanomaterials (Ln = Ce, Eu, Tb and Ho).** A total of 0.03 mmol lanthanide nitrate hydrate was dissolved in 12 mL mixture of DMF and EtOH (v:v=3:1) in a 20 mL capped glass vial. Then 8 mg TCPP dissolved in 4 mL mixture of DMF and EtOH (v:v=3:1) was added dropwise under stirring. After that, the solution was sonicated for 10 min. Then the solution was transferred into a 25 mL Teflon lined autoclave and heated to 90 °C and then kept the reaction for 24 h. The resulting purple results were washed with DI water for 3-5 times and collected by centrifugation at 9500 rpm for 5 min. Finally, the obtained PPF-Ln nanomaterials were freeze-dried.

**Drug loading and releasing.** For DOX loading, PPF-Gd NSs (0.2 mg mL^-1^) were mixed with different concentrations of DOX in phosphate buffer solution (PBS) at pH 7.4. After stirred at room temperature for 24 h, the complexes were collected by centrifugation at 9500 rpm for 5 min and washed by PBS (pH 7.4) for four times to remove excess unloaded DOX. UV-vis spectra of PPF-Gd NSs before and after DOX loading were recorded to determine drug loading ratios. The absorption peaks at 480 nm was used to determine DOX concentrations after the absorbance contribution from PPF-Gd was subtracted from the spectra of PPF-Gd/DOX. The drug loading capacity was calculated according to the following formula:

m(loaded DOX)/m(PPF-Gd)×100%.

To determine drug release kinetics, 1 mL of PPF-Gd/DOX (4mg mL^-1^) was packaged in a dialysis bag (5 KDa) and then immersed within 9 mL PBS at pH 7.4 and pH 5.5 respectively. At different time points, 3 mL of the outside solution was collected and measured by the absorbance spectrometer to determine the concentrations of released drugs, and then 3 mL of PBS at corresponding pH were added to ensure the constant volume. The whole process of drug loading and drug release was performed under dark conditions at 37 °C.

***In vitro* cytotoxicity of PPF-Gd NSs.** Myoblasts (C2C12 cells) obtained from cell bank (Chinese Academy of Sciences, Shanghai, China) were seeded in a 96-well plate (6000 cells per well) and incubated in a humidified atmosphere at 5% CO_2_ and 37 °C. The *in vitro* cell cytotoxicity assessment was carried out by culturing cells with PPF-Gd NSs at different concentrations (5 μg mL^-1^, 10 μg mL^-1^, 30 μg mL^-1^, 50 μg mL^-1^ and 70 μg mL^-1^). After culturing for 24 h, Alamar blue kit (Life Science) was employed to determine the cell viabilities relative to the control cells incubated with the same volume of culture medium (DMEM supplemented with 10% FBS).

**Hemocompatibility analysis of PPF-Gd NSs.** Briefly, to obtain the red blood cells (RBCs), 2 mL of fresh heparin-stabilized blood (6 weeks, female rats, 200-220 g) was added to 10 mL of Dulbecco’s phosphate-buffered saline (D-PBS), followed by centrifugation at 1200 rpm for 10 min. the residual RBCs were washed three times with D-PBS and finally diluted to 20 mL of D-PBS. Then 0.4 mL of RBCs was added to 0,8 mL of PPF-Gd NSs suspension in D-PBS at different concentrations (7.5, 15, 30, 60, 125, 250 and 500 μg/mL). The mixtures of RBCs and nanoparticles were incubated at 37℃ for 1 h and then centrifuged at 1200 rpm for 10 min. 100 μL of supernatant from each sample were transferred to a 96-wells plate and the absorbance at 540 nm was recorded using a microplate reader (SpectraMax@, Molecular Services). RBCs treated with TritonX-100 (2.5%) and D-PBS were used as the positive (+) and negative (-) controls. The percent of hemolysis of RBCs was calculated as follows:

Hemolysis % = (absorbance of sample – absorbance of negative control)/(absorbance of positive control – absorbance of negative control)×%.

**Pharmacokinetics analysis of PPF-Gd NSs.** Four-week-old female Kunming mice were obtained from the Xi’an Jiaotong University. All surgical interventions, treatments and postoperative animal care procedures were performed in strict accordance with the animal care and use guidelines of National Institutes of Health and the animal research committee of Xi’an Jiaotong University. All mice were housed in standard environmental conditions at an ambient temperature of 25 ± 1 °C under a 12 h light-dark cycle. Mice were injected with 20 mg/kg (in terms of mice weight) PPF-Gd nanosheets or saline through tail vein for one time. After treated for 2 d, 5 d, 8d, 11d and 14 d, the mice were sacrificed and the main organs (liver, heart, spleen, lung and kidney) were immediately dissected. Finally, the Gd amount in digestion liquid of the main organs of the mice were measured using ICP machine (7500CE, Agilent).

**Cellular uptake and *in vitro* FL imaging.** Melanoma cells (A375) obtained from cell bank (Chinese Academy of Sciences, Shanghai, China) were used to study the cellular uptake efficiency of the PPF-Gd NSs. Typically, A375 cells were incubated with PPF-Gd NSs (50 μg mL^-1^) for 5 h, 10 h and 24 h, respectively. Cells were washed twice with PBS and fixed with 4% paraformaldehyde solutions before confocal imaging. The cell nucleus was stained by Hoechst33342 (Life Science). The fluorescence of PPF-Gd NSs was excited by a 504 nm laser and observed with the Laser Confocal Microscopy (FV1200, Olympus).

***In vitro* cancer cells therapy.** A375 cells were used to evaluate the *in vitro* chemotherapy effect of PPF-Gd/DOX. Cells were seeded in 96-wells cell culture plate at a density of 3000 cells/well and incubated for 24 h. Then the cells were treated by new medium containing PPF-Gd/DOX with a concentration of 50 μg mL^-1^ and further incubated at 37 °C for another 24 h and 72 h. The cell survival efficiency was measured by using alamar blue kit. PPF-Gd NSs and free DOX were used as controls.

***In vitro* and *in vivo* MR imaging.** The *in vitro* and *in vivo* MR imaging experiments of PPF-Gd NSs were performed in a 0.5 T MRI magnet (MiniMR-60, China). PPF-Gd NSs with different Gd^3+^ concentrations were dispersed in water for the test. All animal experiments were carried out according to protocols approved by Xi’an Jiaotong University Laboratory Animal Center. The *in vivo* MR imaging of NSs was conducted in a tumor-bearing nude mouse model. To develop the tumor model, A375 cells (3×10^6^) suspended in 100 μL of PBS were subcutaneously injected into the back of each female BALB/c nude mouse (5-6 weeks old, purchased from Xi’an KEAO Biotechnology Co., Ltd.). After 15 days injection, the mouse with tumors (about 100 mm^3^) was used for MR imaging ability study. Typically, the mouse was scanned before and after subcutaneous injection and intravenous injection of PPF-Gd NSs for 6h (100 μL, 100 μg mL^-1^), respectively. The obtained relaxation time *T_1_* values were recorded and plotted as 1/*T_1_* versus molar concentrations of Gd (mM).

***In vivo* FL imaging and tumor penetration ability.** For subcutaneous injection, A375 tumor-bearing nude mice were first anesthetized by isoflurane, and PPF-Gd NSs (100 μL, 50 μg mL^-1^) were subcutaneously injected around the tumor tissue. The images of the mice were recorded on an IVIS Spectrum *in vivo* imaging system (LVIS SPECTR, USA) at different time pointes. The mice were sacrificed at 72 h post injection. The main organs (heart, liver, spleen, lung and kidney) and tumors of the mice were harvested and analyzed by the IVIS Spectrum *in vivo* imaging system. Mice treated with PBS were used as control. The main organs (heart, liver, spleen, lung and kidney) and tumors of the mice were collected at different time points (0 h, 24 h and 72 h post injection) and further analyzed by the IVIS Spectrum *in vivo* imaging system. Then the tumor tissues were fixed in 4% (v/v) paraformaldehyde solution, embedded in paraffin, sectioned into 5 μm slices and observed with an inverted fluorescence microscope (Olympus, IX53) to further study the tumor penetration ability of PPF-Gd NSs.

For intravenous injection, PPF-Gd NSs (100 μL, 50 μg mL^-1^) and DOX (100 μL, 150 μg mL^-1^) were injected into the A375 tumor-bearing nude mice, respectively. The FL images of the mice were recorded at different time points (0 h, 24 h, 48 h and 72 h). The mice were sacrificed at 72 h post injection. The main organs (heart, liver, spleen, lung and kidney) and tumors of the mice were harvested and analyzed by the IVIS Spectrum *in vivo* imaging system.

***In vivo* tumor therapy through subcutaneous injection.** A375 tumor-bearing nude mice were randomly divided into four groups (n=5 per group) when the tumor volume reached ~100 mm^3^. Each group of mice was subcutaneously injected with 100 μL of PBS, DOX, PPF-Gd NSs and PPF-Gd/DOX (in terms of DOX 150 μg mL^-1^ and PPF-Gd NSs 50 μg mL^-1^) every other day. The length and width for the tumors were monitored by a digital caliper every other day for 2 weeks. The tumor volume was calculated according to the following formula: width^2^ × length/2.

All the mice were sacrificed on the 14th day and the tumors were collected. The weight and volumes of the dissected tumors were recorded. Then the tumor tissues were fixed in 10% formalin, embedded in paraffin, and sectioned into 4 μm slices for hematoxylin and eosin (H&E, Sigma) staining and TUNEL (Sigma) staining. The liver, spleen, kidney, heart, and lung of the mice were also harvested and fixed for H&E staining. The stained organ and tumor slices were observed under the microscope (Olympus, BX53F).

***In vivo* tumor therapy through intravenous injection.** A375 tumor-bearing nude mice were randomly divided into four groups (n=4 per group) when the tumor volume reached ~100 mm^3^. Each group of mice was intravenously injected with 100 μL of PBS, DOX, PPF-Gd NSs and PPF-Gd/DOX (in terms of DOX 150 μg mL^-1^ and PPF-Gd NSs 50 μg mL^-1^) every other day. The length and width for the tumors were monitored by a digital caliper every other day for 2 weeks. The tumor volume was calculated according to the following formula: width^2^ × length/2.

All the mice were sacrificed on the 14th day and the tumors were collected. The weight and volumes of the dissected tumors were recorded. Then the tumor tissues were fixed in 10% formalin, embedded in paraffin, and sectioned into 4 μm slices for hematoxylin and eosin (H&E, Sigma) staining and TUNEL (Sigma) staining. The liver, spleen, kidney, heart, and lung of the mice were also harvested and fixed for H&E staining. The stained organ and tumor slices were observed under the microscope (Olympus, BX53F).


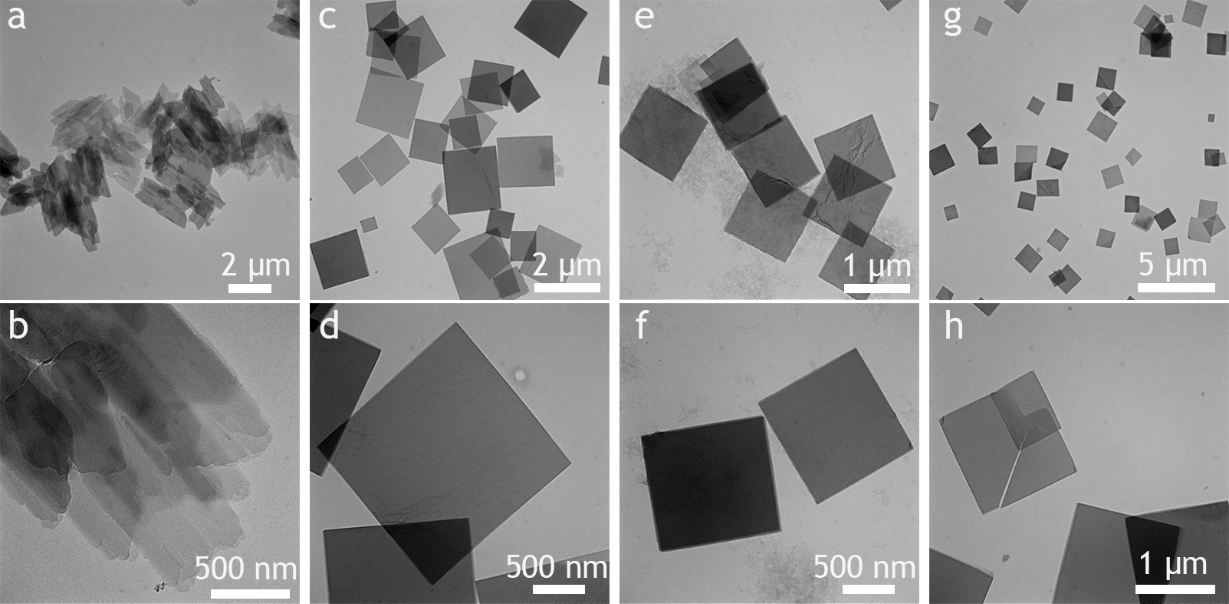


**Figure S1.** TEM images of (a,b) PPF-Ce, (c,d) PPF-Eu, (e,f) PPF-Tb and (g,h) PPF-Ho nanomaterials.


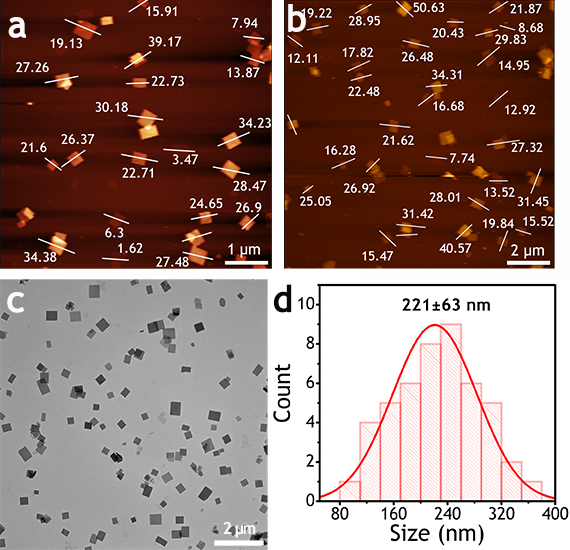


**Figure S2.** (a, b) AFM images of PPF-Gd NSs. (c) TEM image of PPF-Gd NSs. (d) Statistical analysis of the edge length of PPF-Gd NSs measured in TEM image.


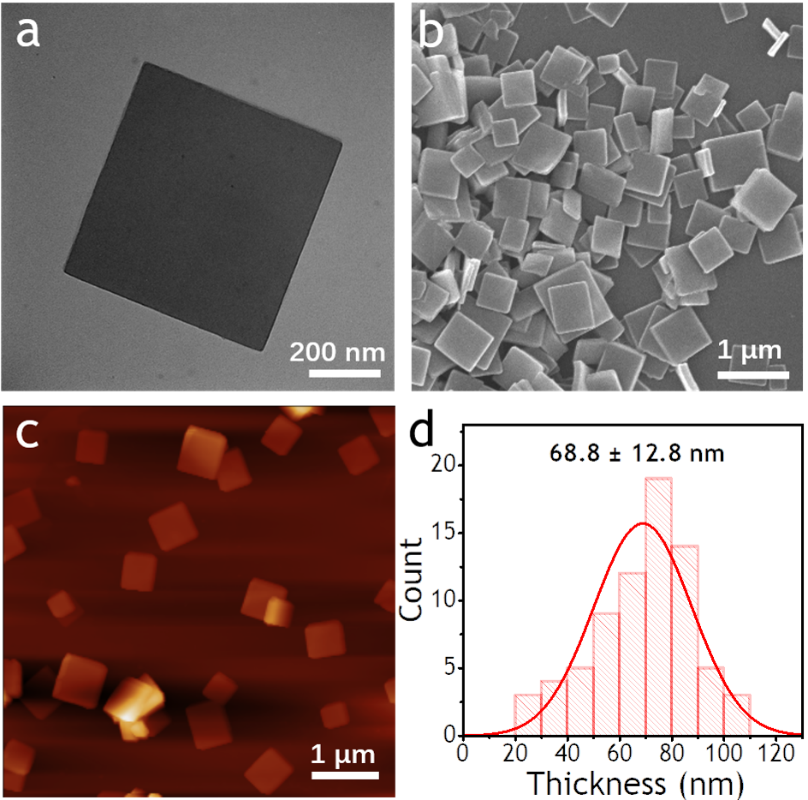


**Figure S3.** (a) TEM image, (b) SEM image and (c) AFM image of PPF-Gd nanoplates. (d) Statistical analysis of the thickness of PPF-Gd nanoplates measured in AFM images.


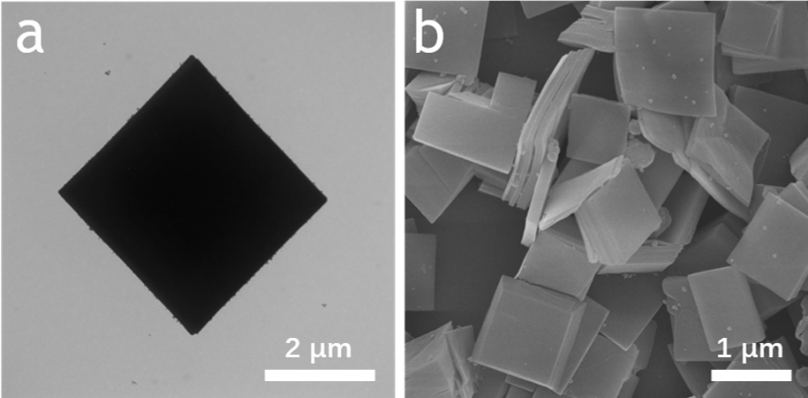


**Figure S4.** (a) TEM image and (b) SEM image of PPF-Gd micro materials.


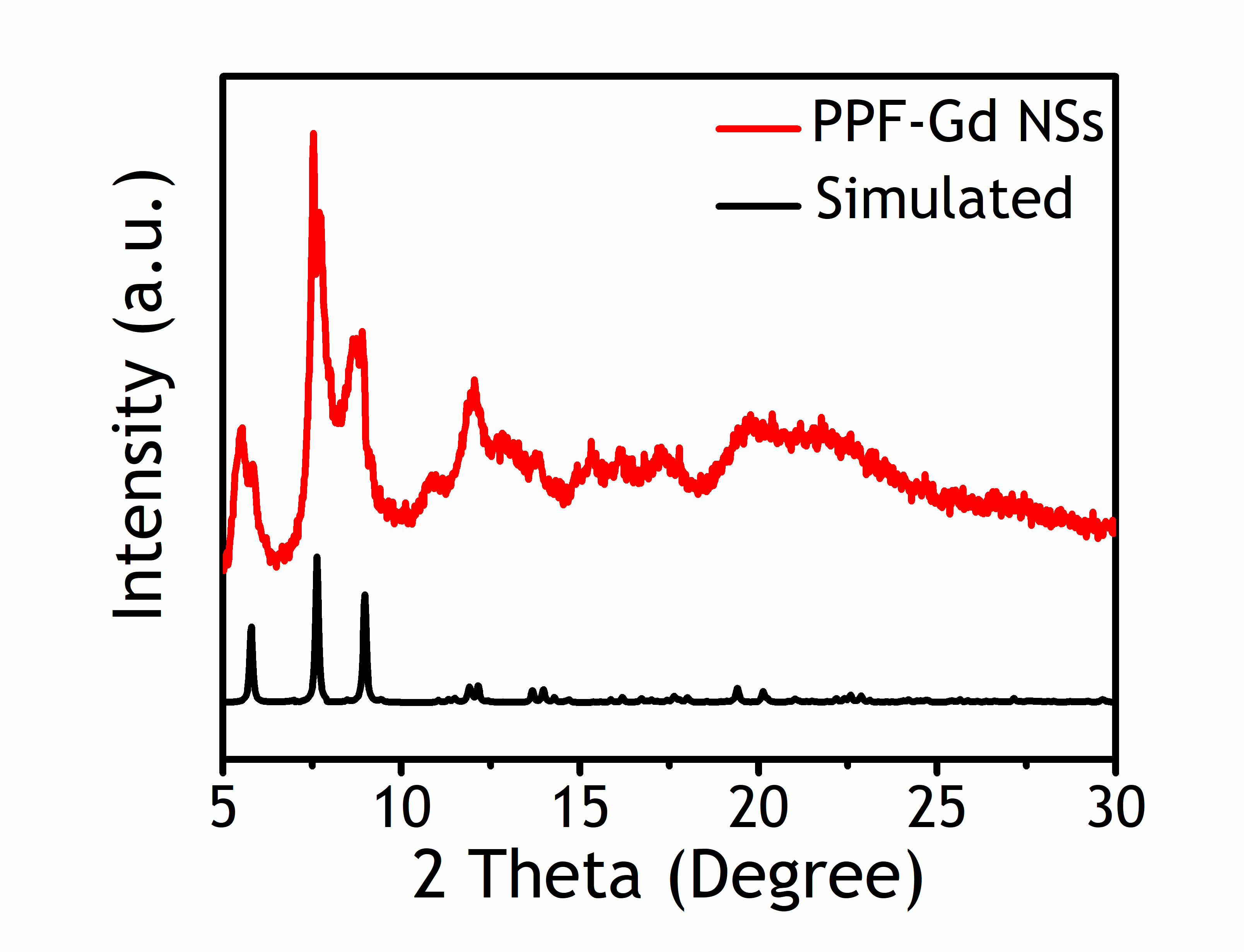


**Figure S5.** XRD pattern of PPF-Gd NSs.


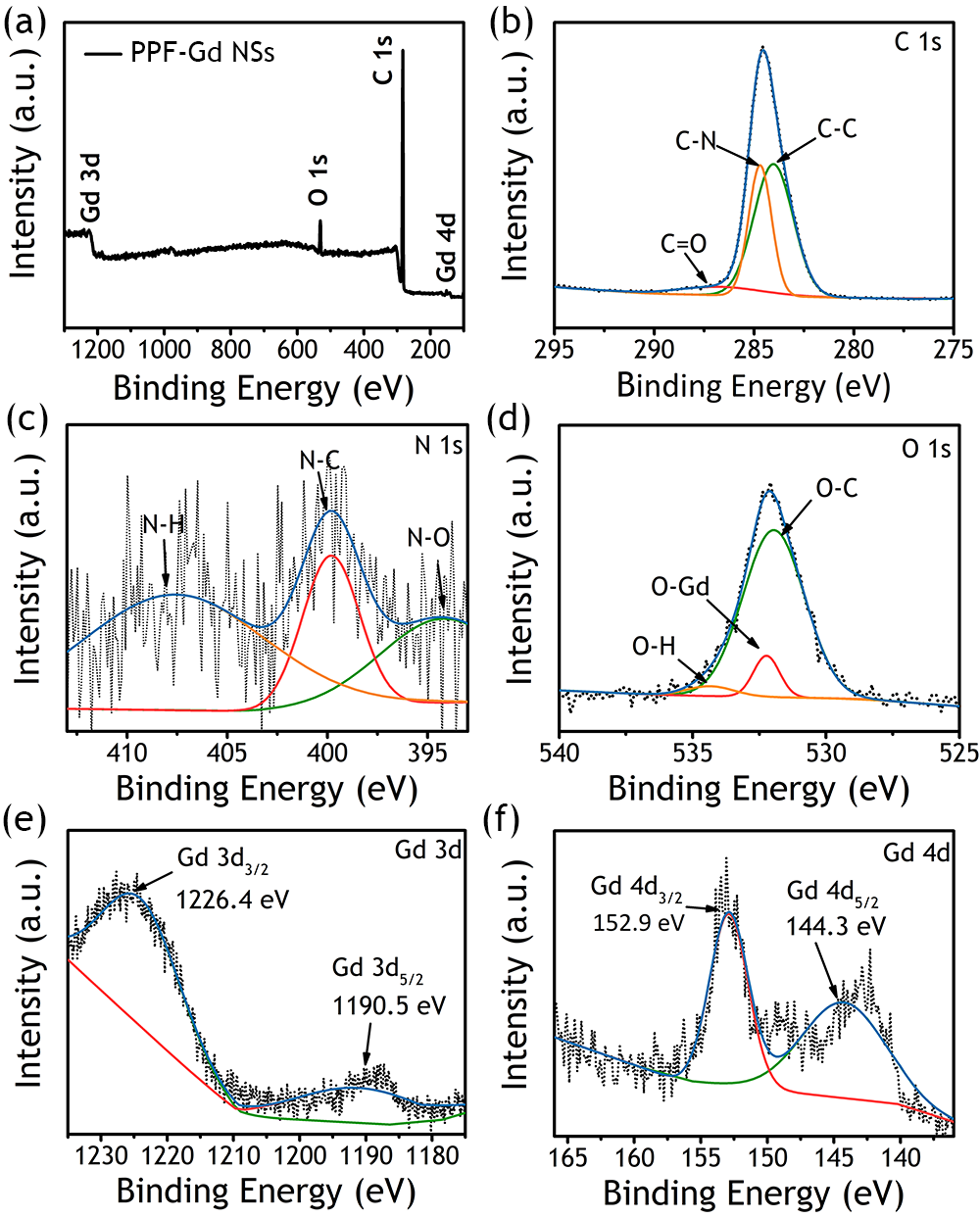


**Figure S6.** High resolution (a) survey, (b) C 1s, (c) N 1s, (d) O 1s, (e) Gd 3d and (f) Gd 4d XPS spectra of PPF-Gd NSs.


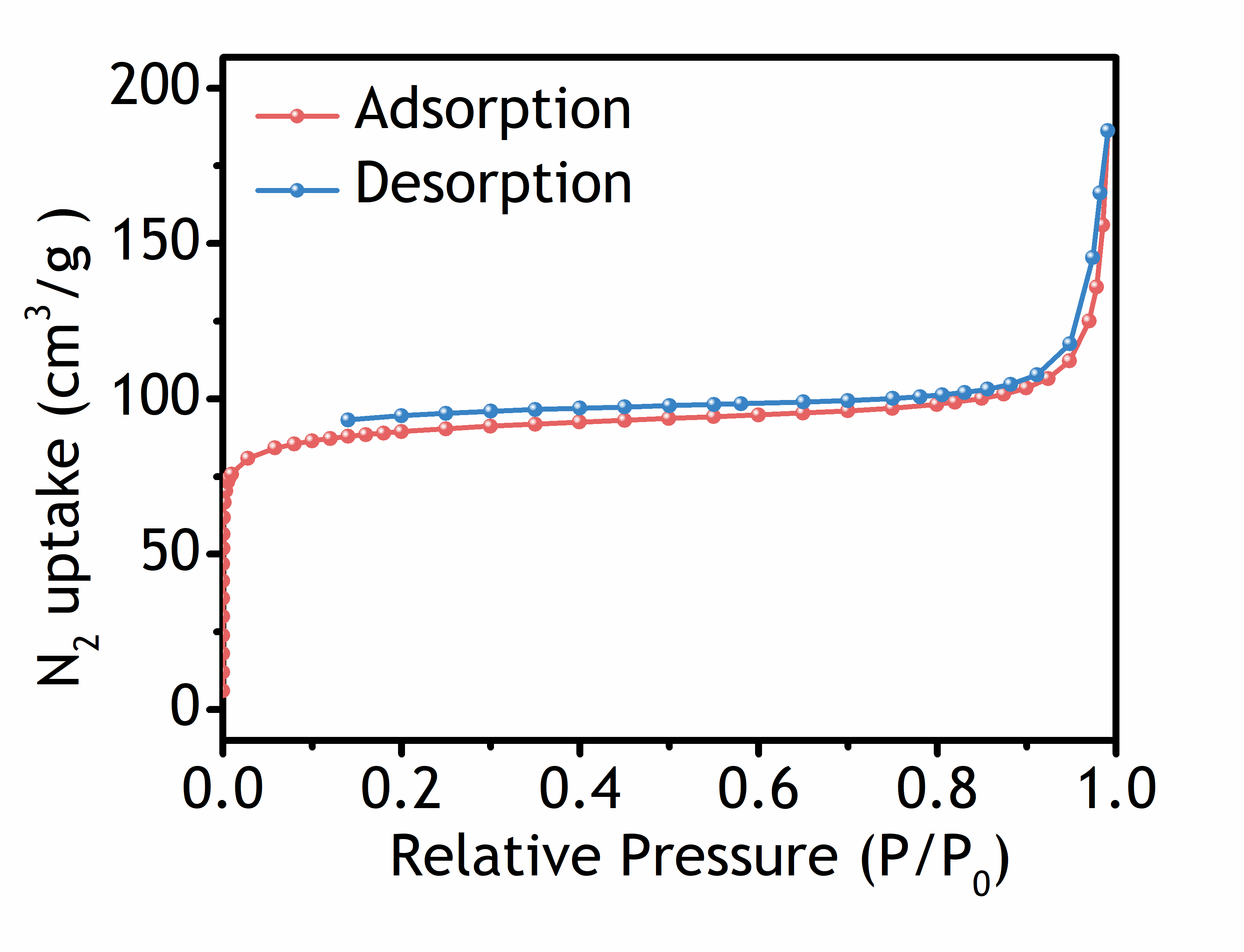


**Figure S7**. N_2_ adsorption isotherms for PPF-Gd NSs at 77 K.


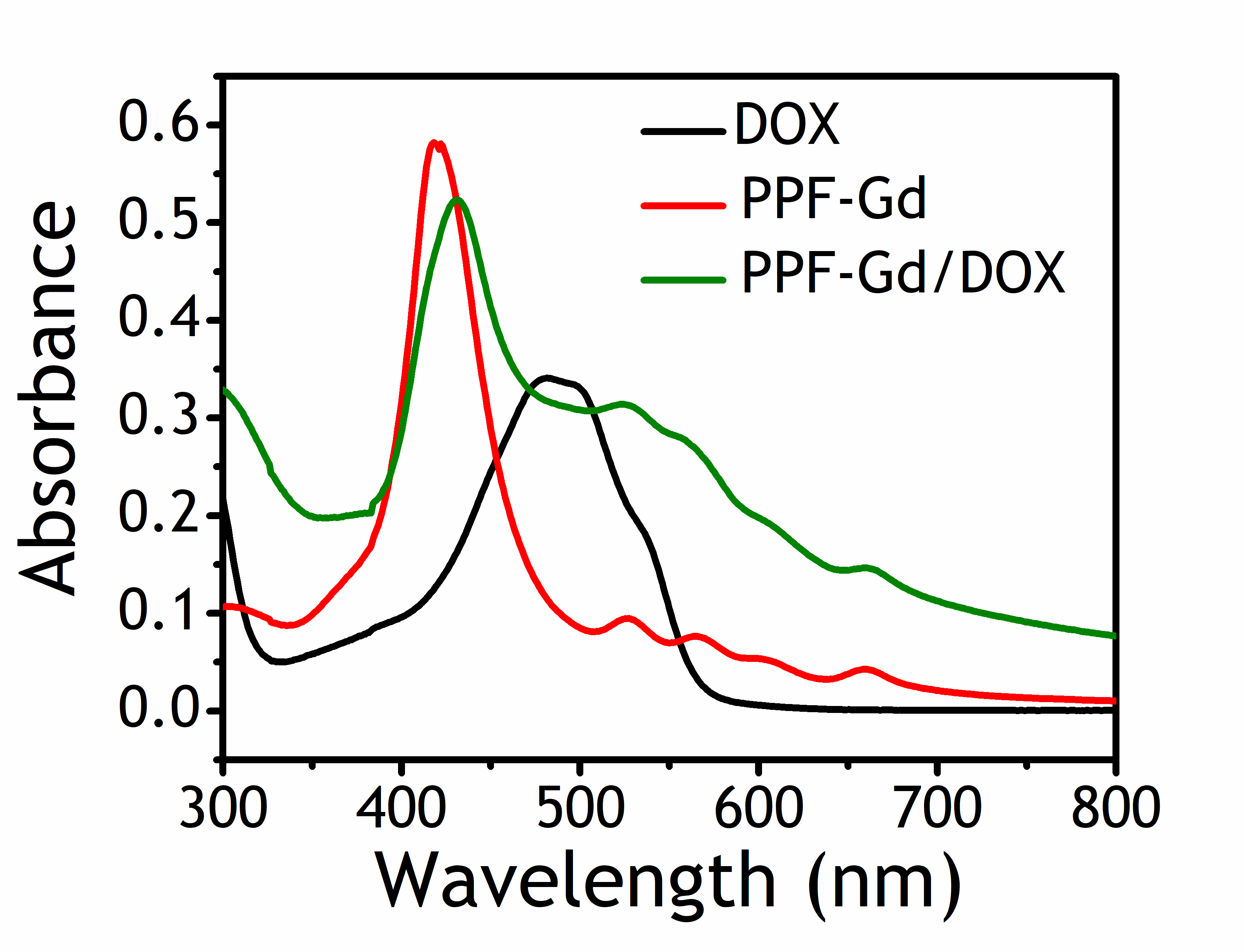


**Figure S8.** UV-vis spectra of DOX, PPF-Gd and PPF-Gd/DOX.


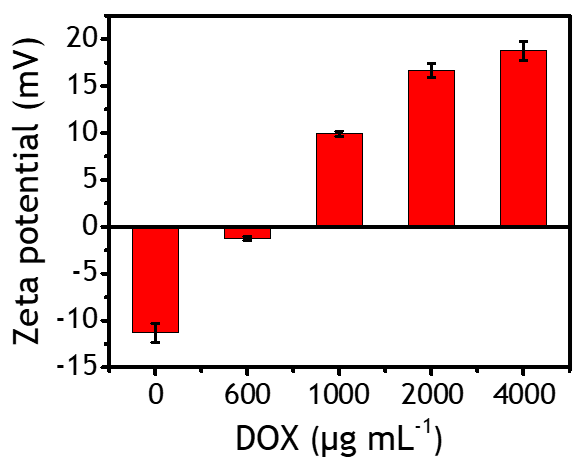


**Figure S9.** Surface zeta-potential of PPF-Gd/DOX with different DOX loading concentrations.


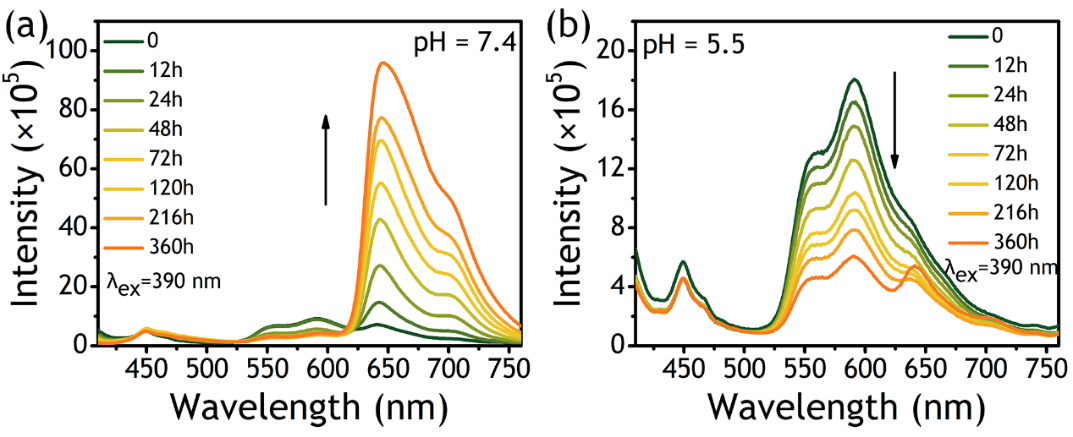


**Figure S10.** The FL spectra of PPF-Gd/DOX during DOX release process at (a) pH 7.4 and (b) pH 5.5.


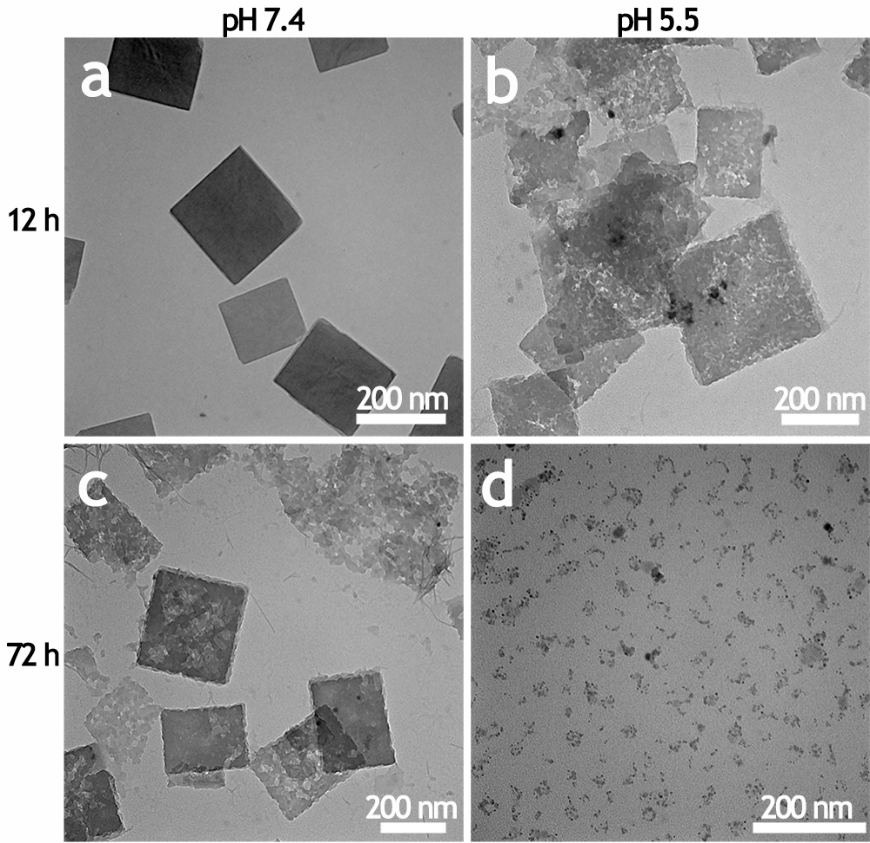


**Figure S11.** TEM images of PPF-Gd NSs at pH 7.4 and pH 5.5 for (a, b) 12 h and (c, d) 72 h, respectively.


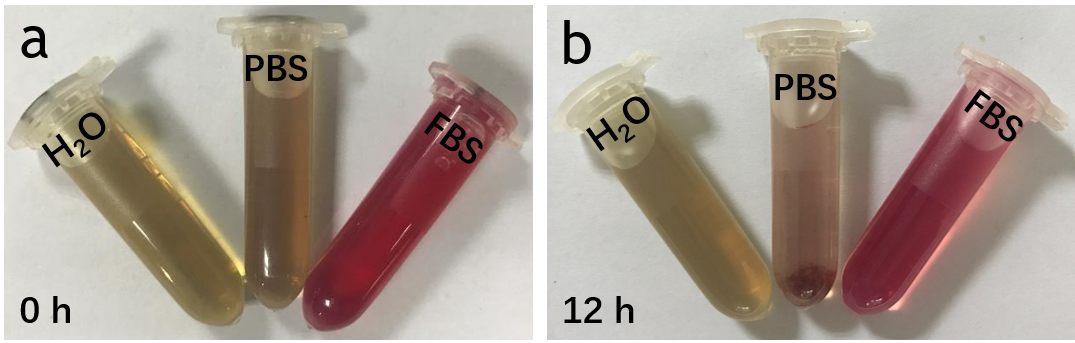


**Figure S12.** Photographs of PPF-Gd NSs in water, PBS (pH 7.4) and serum medium at (a) 0 h and (b) 12h.


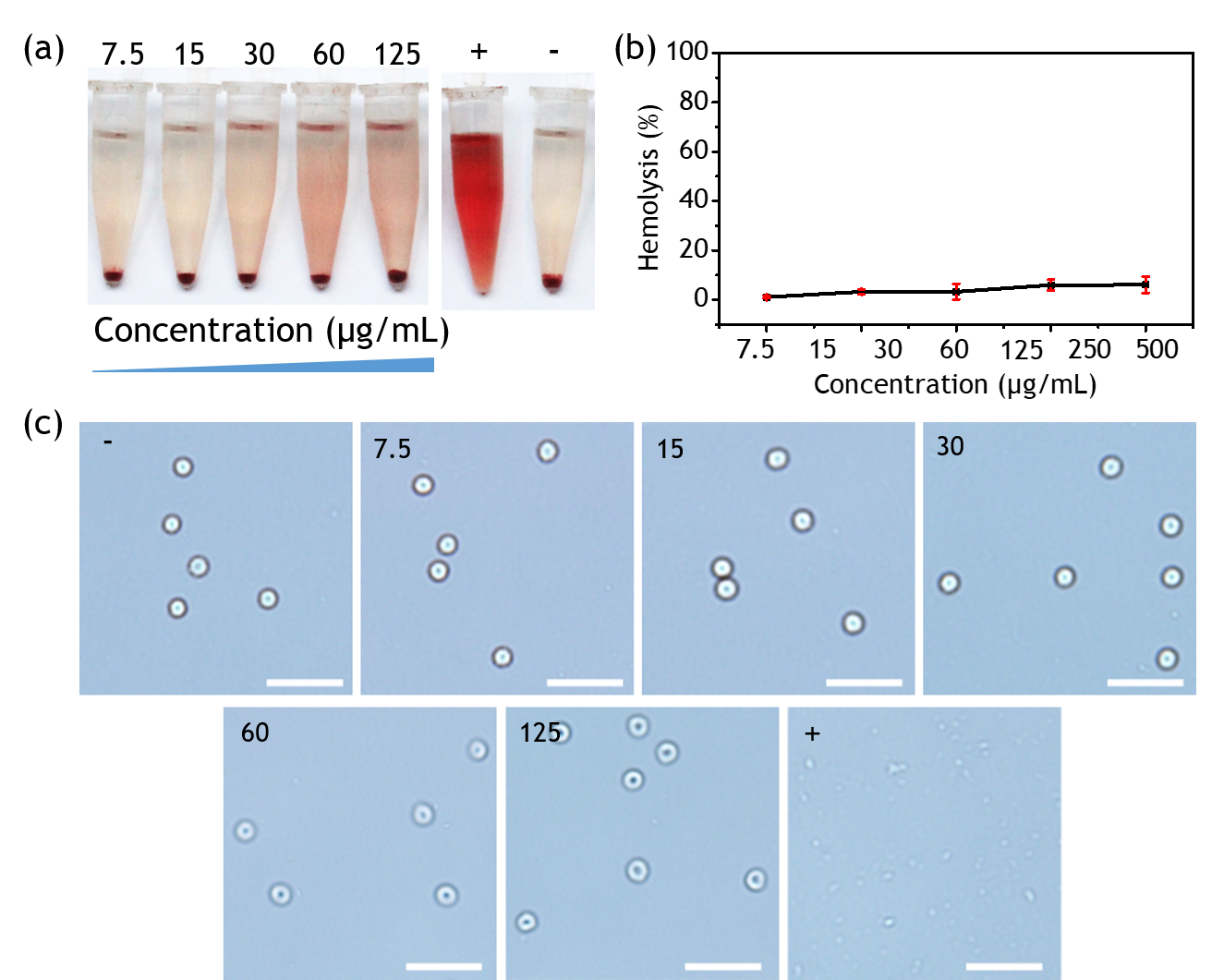


**Figure S13.** Hemolysis effects analysis of PPF-Gd NSs. (a) hemolytic behavior of PPF-Gd NSs at different concentrations with rat RBCs; (b) Hemolysis percentage of RBCs; (c) Optical images of RBC after treated by PPF-Gd NSs with different concentrations, PBS and Triton X-100 were used as negative and positive control. The scale bar = 20 μm.


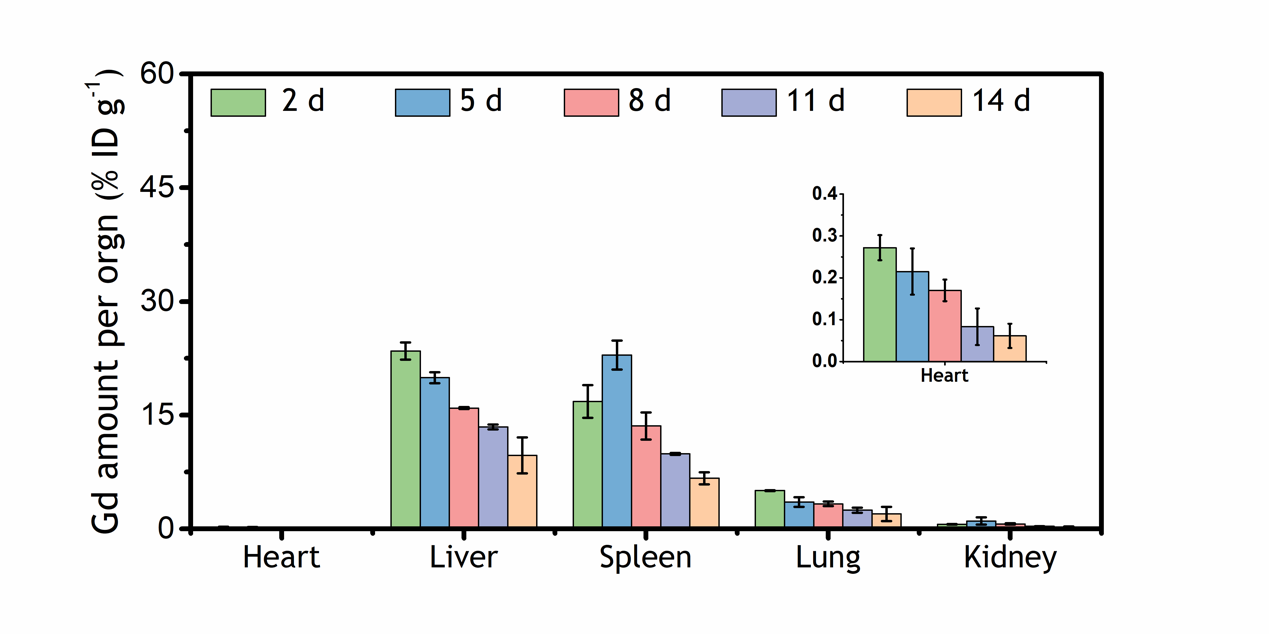


**Figure S14.** Gd contents in the digestion solution of different organs (heart, liver, spleen, lung and kidney) after intravenous injection of PPF-Gd NSs for different time.


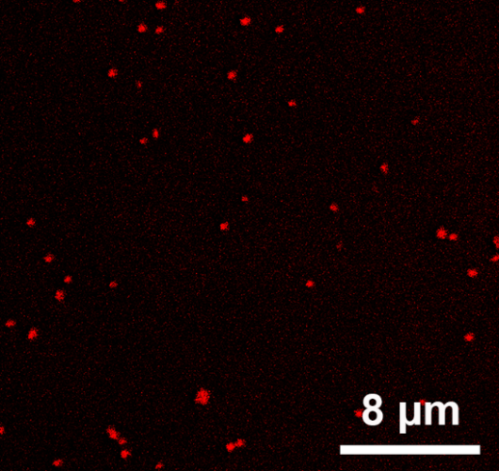


**Figure S15.** Confocal image of PPF-Gd NSs.


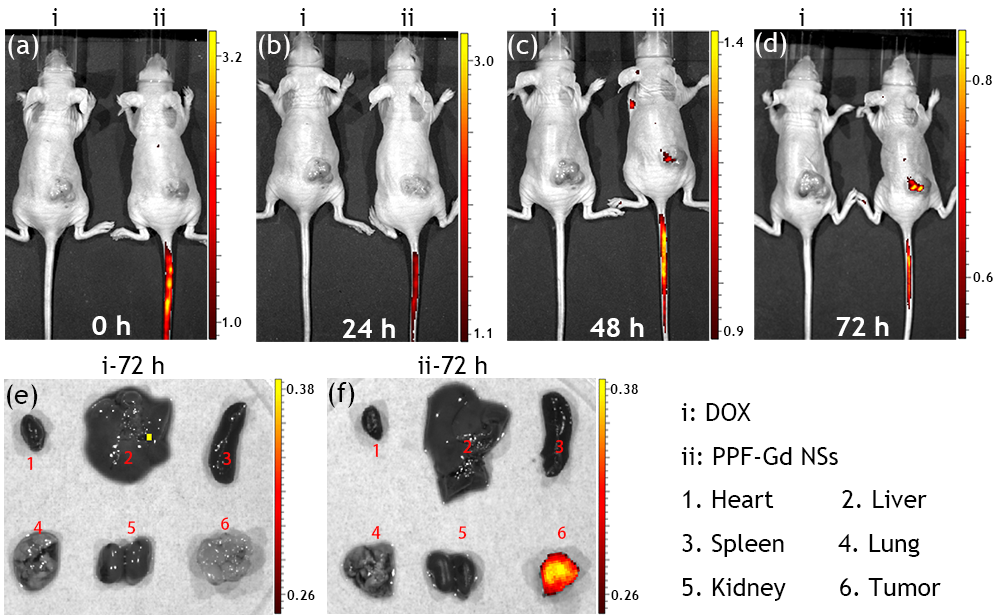


**Figure S16**. (a-d) *In vivo* FL imaging of nude mice bearing A375 tumors at different time point after intravenous injection of PPF-Gd NSs and DOX. (e) FL imaging of major organs and tumor of nude mice after intravenous injection for 72 h of DOX and (f) PPF-Gd NSs. The color scale bar of FL intensity is (×10^7^).

**Figure S17.** Quantitative analysis of the FL intensity from the FL imaging of mice at different time points.


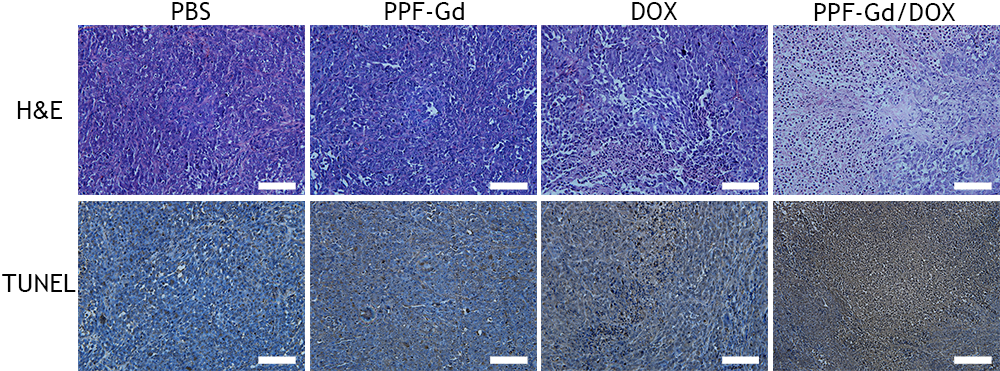


**Figure S18.** Histological microscopy images of the tumor tissues stained with H&E and TUNEL after 14 day treatments through subcutaneous injection. Scale bar = 100 μm.


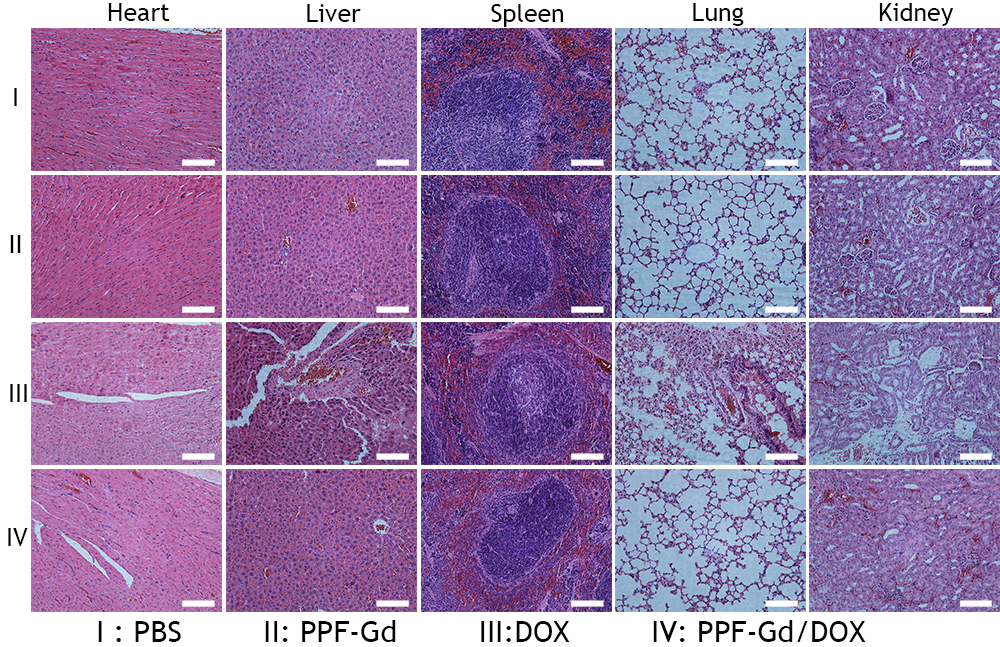


**Figure S19.** Histological microscopy images of the major organs collected on day 14 treated with PBS, PPF-Gd, DOX and PPF-Gd/DOX through subcutaneous injection, respectively. Scale bar = 100 μm.


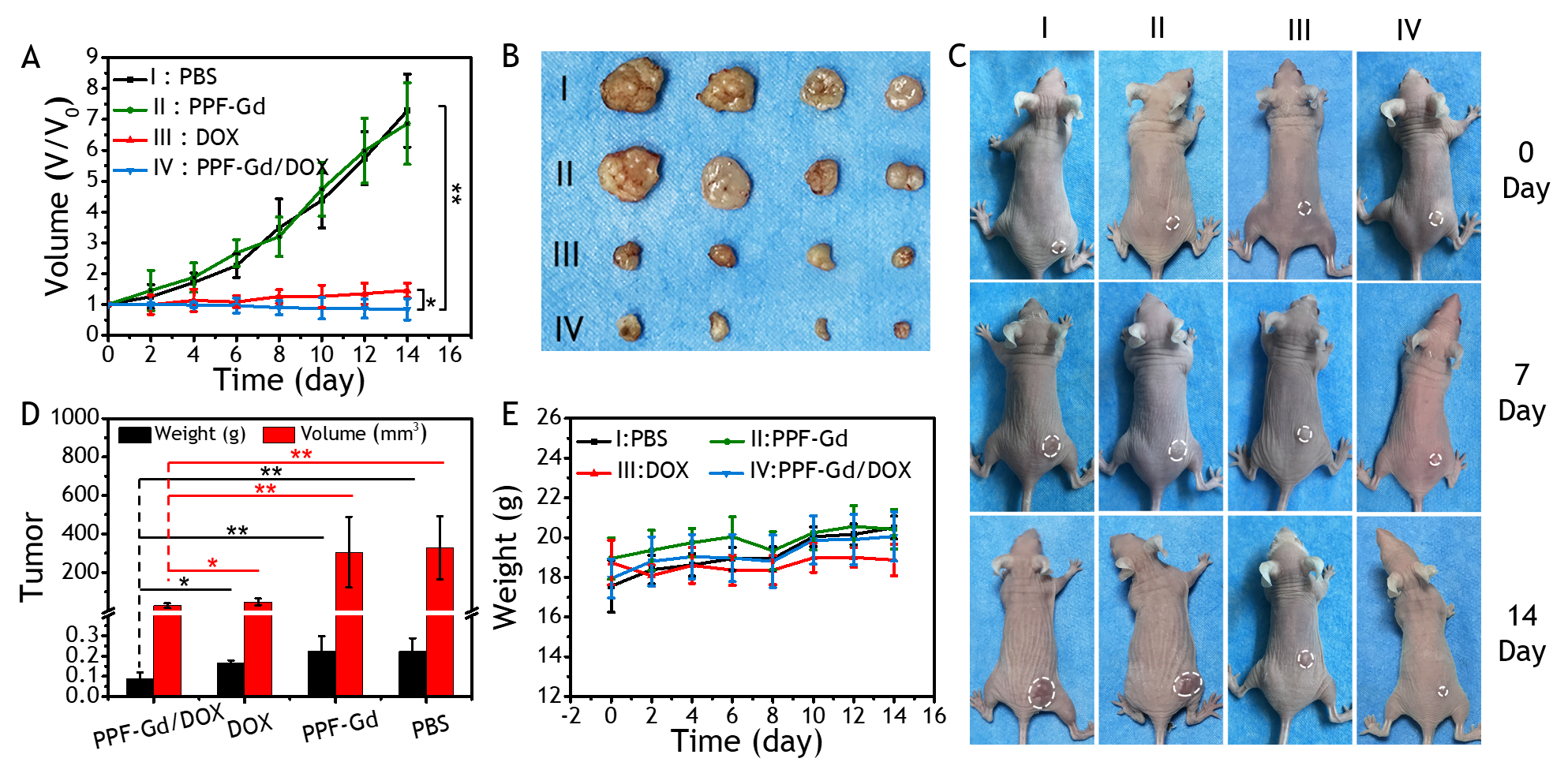


**Figure S20**. (A) Tumor growth curves of A375 tumor-bearing mouse after different treatments through intravenous injection. The tumor volumes were normalized to the initial volumes. (B) Photographs of tumors from different groups after 14-day treatment. (C) Photographs of representative tumors in mice with different treatments. (D) Tumor weights and volumes of each group at the 14th day. (*p<0.05, **p<0.01) (E) The body weights of tumor-bearing mice measured every other day.


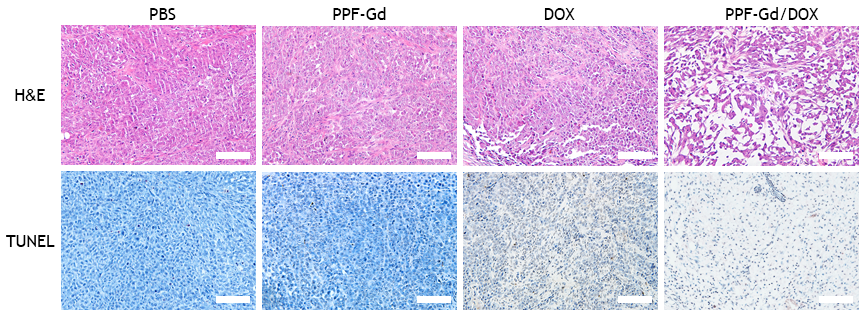


**Figure S21**. Histological microscopy images of the tumor tissues stained with H&E and TUNEL after 14 day treatments through intravenous injection. Scale bar = 100 μm.


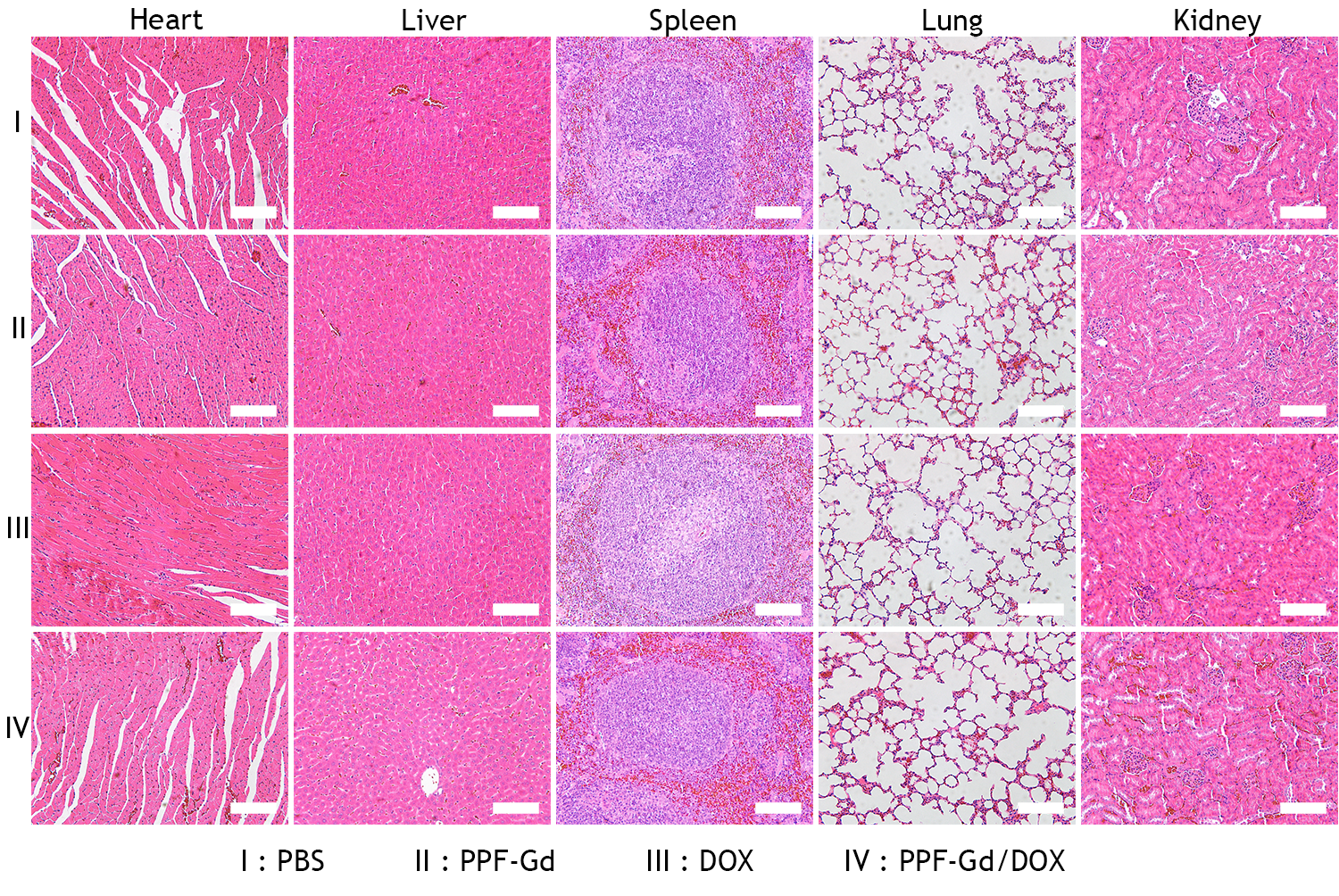


**Figure S22.** Histological microscopy images of the major organs collected on day 14 treated with PBS, PPF-Gd, DOX and PPF-Gd/DOX through intravenous injection, respectively. Scale bar = 100 μm.

**Table S1**. Drug loading capacities of representative 2D nanomaterials and MOF materials.

| **2D nanocarriers** | **Drug** | **Loading Capacity (%)** | **Ref.** |
| --- | --- | --- | --- |
| PPF-Gd NSs | DOX | 300% | This work |
| Lapontite | DOX | 32.8% | [1] |
| WS_2_ | MB | 3.2% | [2] |
| MoS_2_ | DOX | 239% | [3] |
| Fe_3_O_4_@UiO66 | DOX | 196.7% | [4] |
| Zn-CDDB | 5-Fu | 112.8% | [5] |
| ZJU-800 | Diclofenac sodium | 142.7% | [6] |
| ZIF-8 | DOX | 20.0% | [7] |
| MIL-100(Fe) | DOX | 9.9% | [8] |
| PAA@ZIF-8 | DOX | 190.0% | [9] |

**Reference**

1. Li K, Wang S, Wen S, Tang Y, Li J, Shi X, et al. Enhanced in vivo antitumor efficacy of doxorubicin encapsulated within laponite nanodisks. *ACS Appl Mater Interfaces*. 2014; **6**: 12328-34.

2. Yong Y, Zhou L, Gu Z, Yan L, Tian G, Zheng X, et al. WS_2_ nanosheet as a new photosensitizer carrier for combined photodynamic and photothermal therapy of cancer cells. *Nanoscale*. 2014; **6**: 10394-403.

3. Liu T, Wang C, Gu X, Gong H, Cheng L, Shi X, et al. Drug delivery with PEGylated MoS_2_ nano-sheets for combined photothermal and chemotherapy of cancer. *Adv Mater*. 2014; **26**: 3433-40.

4. Zhao HX, Zou Q, Sun SK, Yu C, Zhang X, Li RJ, et al. Theranostic metal-organic framework core-shell composites for magnetic resonance imaging and drug delivery. *Chem Sci*. 2016; **7**: 5294-301.

5. Bag PP, Wang D, Chen Z, Cao R. Outstanding drug loading capacity by water stable microporous MOF: a potential drug carrier. *Chem Commun*. 2016; **52**: 3669-72.

6. Jiang K, Zhang L, Hu Q, Zhao D, Xia T, Lin W, et al. Pressure controlled drug release in a Zr-cluster-based MOF. *J Mater Chem B*. 2016; **4**: 6398-401.

7. Zheng H, Zhang Y, Liu L, Wan W, Guo P, Nystrom AM, et al. One-pot synthesis of metal-organic frameworks with encapsulated target molecules and their applications for controlled drug delivery. *J Amer Chem Soc*. 2016; **138**: 962-8.

8. Horcajada P, Chalati T, Serre C, Gillet B, Sebrie C, Baati T, et al. Porous metal–organic-framework nanoscale carriers as a potential platform for drug delivery and imaging. *Nat Mater*. 2010; **9**: 172.

9. Ren H, Zhang L, An J, Wang T, Li L, Si X, et al. Polyacrylic acid@ zeolitic imidazolate framework-8 nanoparticles with ultrahigh drug loading capability for pH-sensitive drug release. *Chem Commun*. 2014; **50**: 1000-2.
